# Inflammatory protein mediators linking gut microbiota to degenerative lumbar spine disorders: cross-disease genetic evidence

**DOI:** 10.3389/fimmu.2026.1855966

**Published:** 2026-06-03

**Authors:** Yongfu Lou, Daoyi Ma, Qingquan Gan, Xianyue Xu, Yiming Xiao, Junzhuo Wang, Zhibing Li, Tao Zhang, Lei Qi, Shiqing Feng

**Affiliations:** 1 Department of Orthopedics, The Second Qilu Hospital of Shandong University, Jinan, Shandong, China; 2 Shandong University Centre for Orthopaedics, Cheeloo College of Medicine Shandong University, Jinan, Shandong, China; 3 Department of Orthopaedics, Qilu Hospital of Shandong University, Jinan, Shandong, China; 4 Department of Orthopaedics, Tianjin Medical University General Hospital, International Science and Technology Cooperation Base of Spinal Cord Injury, Tianjin Key Laboratory of Spine and Spinal Cord, Tianjin, China

**Keywords:** degenerative lumbar spine disorders, gut microbiota, inflammatory proteins, mediation analysis, Mendelian randomization

## Abstract

**Background:**

Degenerative lumbar spine disorders (DLSD), including intervertebral disc disorders (IDD), degenerative spondylolisthesis, and lumbar spinal stenosis (LSS), are major contributors to low back pain and disability. Associations among gut microbiota (GM), inflammatory proteins, and DLSD have been demonstrated in prior studies. Yet, two key questions persist: whether specific circulating inflammatory proteins (IPs) mediate this association, and whether such mediation is shared across different diseases.

**Methods:**

We performed two-sample Mendelian randomization (MR) to evaluate causal associations among 473 GM taxa, 91 circulating IPs, and three DLSD outcomes using FinnGen R12 summary statistics. Causal estimates were obtained using inverse-variance weighted MR with complementary sensitivity analyses, pleiotropy and heterogeneity testing, and bidirectional MR. Two-step MR mediation was applied to quantify indirect effects of GM through IPs. Experimental validation was performed using rat models, with qPCR and ELISA assessing inflammatory markers in lumbar tissues and metagenomic sequencing evaluating gut microbiota profiles.

**Results:**

Genetically predicted GM taxa were associated with LSS (28 taxa), spondylolisthesis (20 taxa), and IDD (41 taxa). IP MR identified risk-increasing associations for LSS (4E-BP1 and interleukin-4), spondylolisthesis (CXCL1, CXCL5, FGF-5, IL-15RA, and IL-4) and IDD (IL-20RA and IL-6), while IL-18 showed a protective association with IDD that remained robust after multiple-testing correction. Mediation analyses identified 13 genetically supported putative GM–IPs–DLSD pathways, highlighting convergent mediators including PD-L1 for spondylolisthesis and IL-6 and IL-18 for IDD, with mediation proportions ranging from 7.55% to 13.22% across key pathways. Experimental results showed inflammatory activation and gut microbiota alterations in disease models, with partial concordance with the MR findings.

**Conclusions:**

These findings support a genetically determined microbiota–inflammation axis in DLSD. Furthermore, they identify circulating inflammatory proteins as mediators to prioritize mechanistic studies and guide translational follow-up research.

## 1 Introduction

Low back pain (LBP) represents a major global public health challenge and remains the leading cause of years lived with disability worldwide (1, 2). In 2020, LBP affected 619 million people globally. By 2050, that figure is expected to climb to 843 million (1). The key drivers are population ageing and growth. Degenerative lumbar spine disorders (DLSD) constitute a principal etiologic group underlying LBP and associated disability, encompassing intervertebral disc disorders (IDD), degenerative spondylolisthesis (DS), and lumbar spinal stenosis (LSS) (2). Lumbar disc herniation, frequently classified within disc disorders, contributes substantially to radiculopathy, healthcare utilization, and work-related disability (3). With population ageing, the prevalence and clinical impact of degenerative lumbar conditions continue to increase. Degenerative LSS is notably common among the public. According to a comprehensive systematic review and meta-analysis, the average prevalence of this clinically diagnosed condition is estimated at roughly 11% (4). LSS is also a common indication for specialist assessment and intervention in older adults (5). A large community-based study of elderly Chinese adults found lumbar spondylolisthesis in 25.0% of women and 19.1% of men (6). More recent longitudinal observations further support the view that degenerative spondylolisthesis is embedded within a broader degenerative cascade of the functional spinal unit (7). These three phenotypes were studied together because they are linked by the degenerative cascade of the functional spinal unit and share overlapping inflammatory and remodeling processes, even though their dominant anatomical manifestations differ.

Accumulating evidence supports an interaction between the gut microbiota (GM), systemic immune signaling, and musculoskeletal health, forming a gut-immune-musculoskeletal axis (8). The composition of the gut microbiome and its metabolic products can shape systemic inflammatory tone, immune cell activation, and how pain is processed. In this way, they can contribute to chronic pain and degenerative changes in tissues (9, 10). In parallel, inflammatory pathways are central to the pathophysiology of degenerative lumbar disorders. In disc-related disease, inflammatory mediators contribute to extracellular matrix breakdown, cell senescence, and nociceptive sensitization (11, 12). A systematic review further supported an association between interleukin-6 and intervertebral disc degeneration, and intradiscal interleukin-6 has been associated with early postoperative outcomes in lumbar disc disease (13, 14). In LSS, inflammatory and profibrotic signaling participates in ligamentum flavum hypertrophy and canal narrowing (15). Histological and biomechanical studies likewise support chronic inflammatory remodeling as a component of ligamentum flavum hypertrophy (16). In DS, interleukin-19 and interleukin-20 signaling has been implicated in inflammatory responses involving disc and facet tissues associated with segmental instability (17). In addition, cytokine and chemokine profiling studies have supported a broader inflammatory remodeling milieu in lumbar degenerative spondylolisthesis and degenerative facet tissues (18, 19).

From a disease-biology perspective, studying these three lumbar phenotypes within a common framework is justified because they are not isolated entities. Advanced disc degeneration alters segmental mechanics, promotes facet joint overload and ligamentum flavum remodeling, and can ultimately contribute to both stenotic change and instability-related phenotypes (7). Degenerative spondylolisthesis and lumbar spinal stenosis frequently coexist and share structural hallmarks such as canal narrowing, facet degeneration, ligamentous hypertrophy, and chronic inflammatory remodeling (5, 16). This overlap suggests that at least part of the biological susceptibility may be shared across phenotypes rather than being entirely disease-specific.

The same logic applies to inflammation. Although the dominant anatomical substrate differs among IDD, DS, and LSS, available evidence indicates that all three involve persistent low-grade inflammatory activation, extracellular matrix degradation, fibrocartilaginous remodeling, and dysregulated cytokine signaling (11, 12). In disc-related disease, inflammatory mediators contribute to matrix breakdown and pain sensitization (11). In LSS, inflammatory and profibrotic signaling participates in ligamentum flavum hypertrophy and canal narrowing (15, 16). In DS, inflammatory cytokines together with interleukin-19/interleukin-20 signaling have been implicated in facet and disc degeneration associated with segmental instability (17). Additional evidence from cytokine profiling and degenerative facet tissue studies further supports an inflammatory remodeling environment in lumbar degenerative spondylolisthesis (18, 19). Examining inflammation across all three phenotypes is therefore necessary to distinguish shared immune mechanisms from phenotype-specific downstream manifestations.

A similar knowledge gap exists for the gut microbiota. Published studies have begun to associate dysbiosis or genetically predicted microbial taxa with individual lumbar phenotypes, including intervertebral disc degeneration, back pain, and lumbar spinal stenosis (20–22). Conceptual reviews have also highlighted the plausibility of a gut–musculoskeletal axis in degenerative lumbar disorders (23). However, these reports have largely evaluated single diseases or pairwise links, making it difficult to determine whether the same microbial signals recur across major degenerative lumbar phenotypes or whether disease-specific associations predominate.

However, traditional epidemiological research faces major methodological hurdles and reverse causation, complicating causal inference regarding the roles of GM and inflammatory mediators in lumbar degeneration. Mendelian randomization leverages germline genetic variants as instrumental variables under explicit assumptions to reduce confounding and mitigate reverse causation (24, 25). Two-sample designs improve statistical efficiency by combining summary statistics from independent datasets (26, 27). Weighted median and related sensitivity approaches further strengthen robustness when some instruments may be invalid (28). Two-step and multivariable mediation frameworks provide a principled approach to quantify indirect effects through intermediate molecular traits (29, 30). Simple exposure-outcome MR can identify whether gut microbiota are associated with DLSD risk, but it cannot determine whether circulating inflammatory proteins lie on the intermediate pathway between them.

Importantly, the advantage of a mediation framework is not merely statistical. In a setting such as degenerative lumbar disease, where microbial exposures are distal and tissue phenotypes are heterogeneous, mediation analysis helps clarify whether circulating inflammatory proteins may represent biologically meaningful intermediates rather than parallel correlates (30). This is particularly valuable when the same microbial taxa may map onto different clinical phenotypes, because it enables prioritization of shared inflammatory nodes that may have greater mechanistic and translational relevance than isolated taxon–disease associations alone (29). Taken together, the current literature supports the need for an integrated framework that simultaneously considers GM, inflammatory mediators, and multiple degenerative lumbar outcomes. No established consensus set of shared microbial taxa or shared inflammatory proteins has yet been defined across IDD, DS, and LSS. A unified analysis is therefore required not only to estimate disease-specific effects but also to test whether common microbiota–inflammation signatures underlie different manifestations of lumbar degeneration.

Although Mendelian randomization provides genetic evidence for potential causal relationships, it does not directly reflect molecular changes at the tissue level. Therefore, experimental validation is necessary to determine whether the identified inflammatory mediators and microbiota-related signals are biologically manifested in lumbar tissues (31).

In this study, we applied a unified genetic framework integrating gut microbial taxa, circulating inflammatory proteins, and three degenerative lumbar spine disorders from FinnGen R12. We aimed to estimate genetically supported associations between GM and each lumbar outcome, evaluate causal links between inflammatory proteins and disease risk, and quantify inflammatory protein-mediated effects that may connect GM to lumbar degeneration. We further performed *in vivo* validation using rat models, combined with qPCR, ELISA, and gut microbiota sequencing, to bridge the gap between genetic inference and biological evidence.

## 2 Methods

### 2.1 Mendelian randomization

#### 2.1.1 Study design

**Figure 1:**
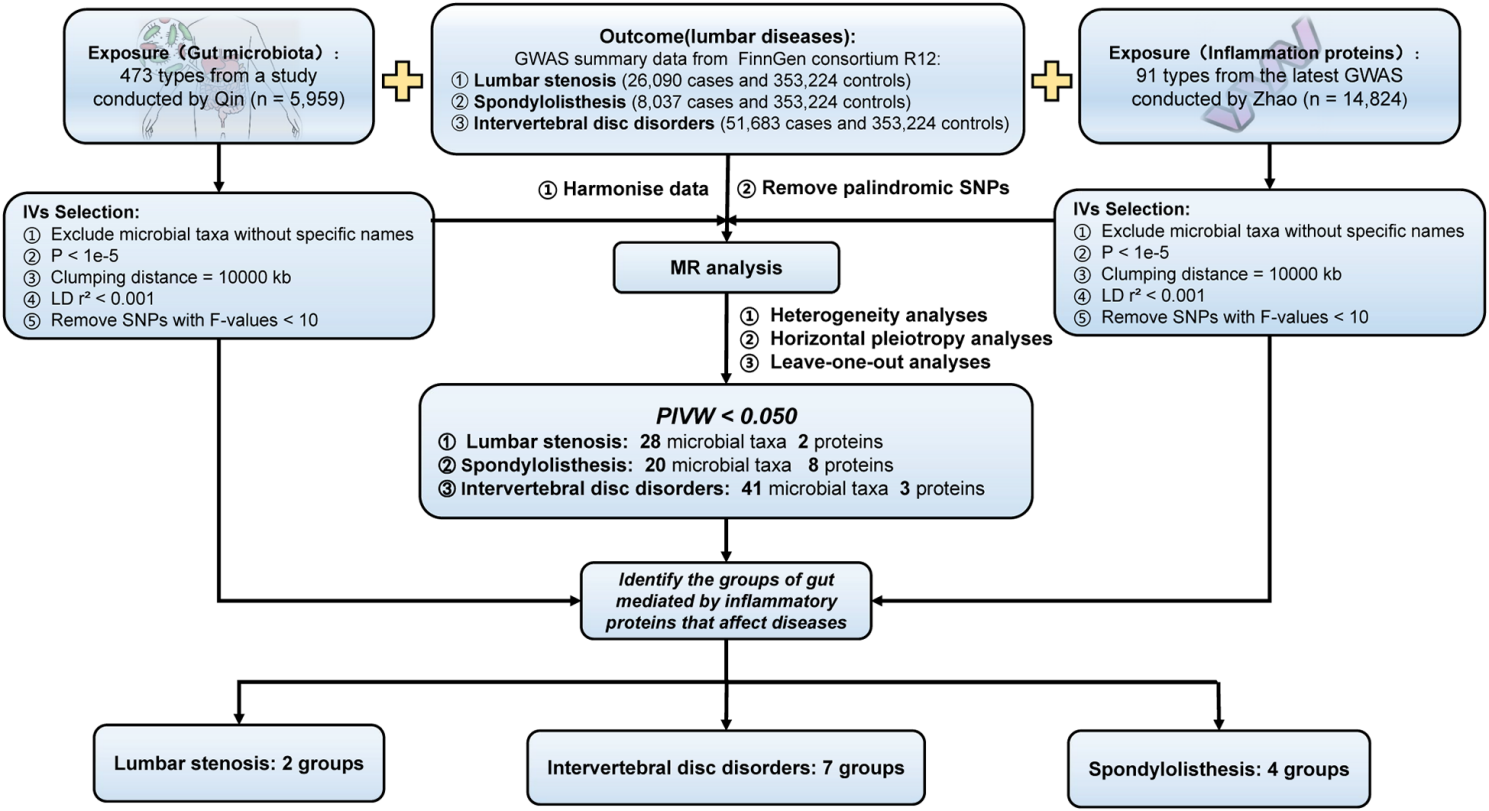
Study design and analytic workflow. Two-sample Mendelian randomization (MR) assessed causal effects of gut microbial on lumbar diseases, followed by MR of circulating inflammatory proteins and two-step mediation to quantify indirect effects; 13 putative microbiota–inflammatory protein–lumbar diseases pathways were identified. SNP, single nucleotide polymorphism; LD, linkage disequilibrium; IV, instrumental variable; IVW, inverse-variance weighted.

**Figure 1** illustrates the flow of our study design. The work is built around three main parts: (1) assessing how 473 gut microbiota (GM) causally affect three lumbar diseases; (2) evaluating the causal influence of 91 inflammatory proteins (IPs) on the same three lumbar conditions; and (3) conducting mediation analysis to clarify how inflammatory proteins serve as intermediaries between gut microbiota and lumbar diseases. For two−sample Mendelian Randomization (MR), we followed three core assumptions to reduce bias: (1) instrumental variables (IVs) need to be strongly linked to the exposure factors; (2) IVs should be independent of any confounders; (3) there must be no direct relationship between the IVs and the outcome (25).We followed the Strengthening the Reporting of Observational Studies in Epidemiology using Mendelian Randomization (STROBE-MR) when reporting this study.

#### 2.1.2 Data sources

Genetic data for gut microbiota were derived from the work by Qin and colleagues. This study analyzed data from 5,959 individuals. All had genotype-matched gut microbiome (GM), diet, and health records. The analysis revealed 567 independent SNP-taxon associations revealing the role of human genetic variation in shaping GM abundance (32). Genetic data for circulating inflammatory proteins were obtained from a comprehensive genome-wide protein quantitative trait locus (pQTL) analysis covering 91 plasma proteins. Protein measurements were generated using the Olink Target platform in a cohort of 14,824 participants. Protein levels were quantified in Normalized Protein eXpression (NPX) units, and MR estimates represent per-standard deviation (SD) changes (33). Summary statistics for lumbar stenosis, spondylolisthesis and intervertebral disc disorders were obtained from the FinnGen Consortium R12 dataset, which was released in November 2024 and has a sample size of 500,348, including 282,064 females and 218,284 males (34)(**Table 1**).

**Table 1:** Characteristics of GWAS summary statistics used as exposure (taxa), mediator (inflammatory proteins), and outcomes (lumbar diseases).

Phenotypes	Phenotypic source	Data source	Sample size (case/controls)	Ancestry
Exposure				
Gut microbiota	GCST90032172-GCST90032644	IEU openGWAS	5,959	European
Inflammatory proteins	GCST90274758-GCST90274848	GWAS Catalog	14,824	European
Outcome				
Lumbar stenosis	M13_SPINSTENOSIS	FinnGen R12	379,314 (26,090/353,224)	European
Spondylolisthesis	M13_SPONDYLOLISTHESIS	FinnGen R12	361,261 (8,037/353,224)	European
Intervertebral disc disorders	M13_INTERVERTEB	FinnGen R12	404,907 (51,683/353,224)	European

This table shows the specific data information of gut microbiota, inflammatory proteins, and lumbar diseases. Values denote total sample sizes and, for outcomes, case/control counts. All datasets are of predominantly European ancestry. Genetic variants linked to the gut microbiome were identified through genome-wide association analysis of 7,979,834 variants derived from 5,959 individuals in the Finnish FINRISK 2002 cohort. Lumbar disease endpoints (Lumbar stenosis, Spondylolisthesis, Intervertebral disc disorders) were obtained from FinnGen R12 (endpoint IDs M13_SPINSTENOSIS, M13_SPONDYLOLISTHESIS, M13_INTERVERTEB).

#### 2.1.3 Instrumental variables selection

First, single nucleotide polymorphisms (SNPs) significantly associated with exposure factors (P < 1×10^–5^) were chosen. Second, the linkage disequilibrium (LD) of these SNPs had to satisfy r^2^ < 0.001 and a clumping distance of 10 Mb. Third, the statistical significance of SNPs was evaluated using the formulae F = β^2^/se^2^ and R^2^ = 2 × (1- MAF) × MAF × β^2^, with SNPs showing weak associations (F < 10) were excluded (35). Clumping was performed at r^2^<0.001 within 10 Mb using a European LD reference panel. Instrument strength per exposure was summarized by F-statistics (minimum F > 10) and variance explained (R^2^). Fourth, palindromic SNPs were also removed to ensure consistent allele impacts on exposure and outcomes.

#### 2.1.4 Causal effect estimation and sensitivity analyses

We used five MR methods in a bidirectional analysis to explore causal relationships among gut microbiota, inflammatory proteins, and lumbar diseases: namely Inverse Variance Weighted (IVW), weighted median, weighted mode, simple mode, and MR-Egger regression, with IVW being the principal method (36). Extensive sensitivity analyses were also conducted. Cochran’s Q test was applied to assess heterogeneity, and the MR-Egger intercept and MR-PRESSO global test were used as diagnostic tools to detect potential horizontal pleiotropy rather than to exclude it completely (37). For sensitivity analysis, we employed the”leave-one-out”approach to ensure that no single SNP significantly altered the overall estimate (38). We applied the Benjamini-Hochberg method to correct for multiple comparisons among gut microbiota and inflammatory proteins, and defined a causal relationship as significant when the false discovery rate (FDR) was below 0.05. All the statistical analyses were performed via R (version 4.4.1). The “TwoSampleMR” package (version 0.6.5) and the “MR-PRESSO” package (version 1.0) were employed to conduct the MR and MR-PRESSO analyses, respectively.

To investigate the bidirectional causal relationship, we used lumbar diseases as the “exposure” and gut microbiota or inflammatory proteins linked to these diseases as the “outcome”. SNPs significantly associated with lumbar diseases (P < 5×10^-8^) were selected as IVs. Given case counts and instrument availability, reverse-MR power was limited for certain outcomes.

#### 2.1.5 Mediation analysis

Mediation analysis was conducted to estimate whether inflammatory proteins may represent putative intermediate traits linking gut microbiota to lumbar diseases. This helps explore potential mechanisms of how exposure impacts outcomes. To estimate the mediation effect proportion, we divided the indirect effect by the total effect, with a 95% confidence interval attached.

### 2.2 Animal experimentation

#### 2.2.1 Establishment of animal models

**Figure 2:**
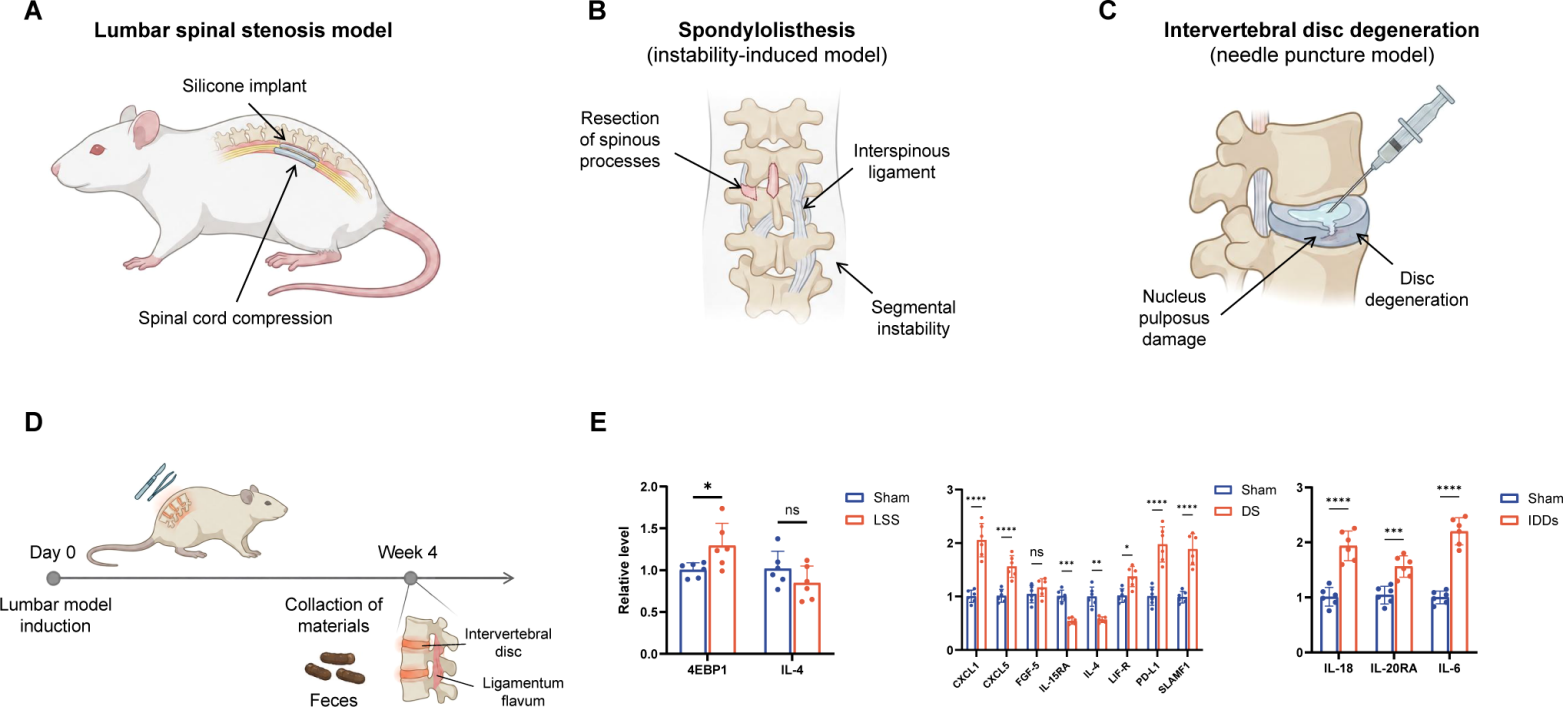
Schematic of rat lumbar spine models, experimental design, and inflammatory gene expression. **(A)** LSS: silicone implant-induced cauda equina compression. **(B)** Spondylolisthesis: ligament resection-caused segmental instability. **(C)** IDD: needle puncture-induced nucleus pulposus damage. **(D)** Timeline: surgery, fecal sampling, lumbar tissue collection. **(E)** Relative mRNA expression levels of inflammatory proteins were quantified using qRT-PCR. (*p < 0.05, **p < 0.01, ***p < 0.001,****p < 0.0001, one-way ANOVA on ranks post Dunn’s Method).

We used healthy, specific pathogen-free (SPF) grade Sprague-Dawley (SD) rats aged 6 to 8 weeks for all *in vivo* surgical experiments. All rats were housed in a controlled environment with a 12h light/dark cycle, constant temperature of 22°C, and relative humidity of 45-60%. For the lumbar spinal stenosis cohort, a chronic cauda equina compression model was established by epidural silicone implantation at the lumbar level. Briefly, after exposure of the lumbar spinal canal by laminotomy, a custom-made silicone block was inserted into the epidural space to produce sustained compression of the cauda equina and adjacent neural structures (39). For the spondylolisthesis cohort, a lumbar instability-induced model was established in rats to mimic degenerative spondylolisthesis-related pathology. Briefly, a posterior approach was used to expose the lumbar spine, and the spinous processes together with the supraspinous and interspinous ligaments were resected at the target segment to disrupt posterior stabilizing structures and induce segmental instability. In selected protocols, partial facet joint resection may be performed to further enhance instability. This model leads to abnormal segmental motion, progressive disc degeneration, ligamentum flavum remodeling, and vertebral translation, thereby recapitulating key features of instability-associated spondylolisthesis (40). For the intervertebral disc disease cohort, a rat lumbar intervertebral disc injury model was established using a published puncture-based lumbar disc model with controlled disc herniation (41). In brief, the target lumbar intervertebral disc was exposed under anesthesia and injured by a standardized puncture procedure to induce disc pathology. At 3 weeks after model establishment, rats were sacrificed, and lumbar tissues were harvested (**Figure 2**).

#### 2.2.2 Quantitative polymerase chain reaction

For the lumbar spinal stenosis and spondylolisthesis groups, the ligamentum flavum from the operated segment was collected as the primary tissue for qPCR analysis. For the intervertebral disc disease group, the operated intervertebral disc tissue was harvested for RNA extraction. Total RNA was extracted using a TRIzol-based method, and its concentration and purity were determined using a NanoDrop spectrophotometer (Thermo Fisher Scientific, USA). Complementary DNA (cDNA) was synthesized from equal amounts of RNA using a reverse transcription kit (Bio-Rad, USA). Quantitative PCR was performed using a detection system (Roche, Switzerland). GAPDH was used as an internal control. Detailed primer sequences are provided in **Table 2**. For normally distributed data, comparisons between groups were performed using independent-samples t-tests or one-way analysis of variance (ANOVA). Statistical significance was defined as a two-tailed p-value < 0.05, and results were expressed with corresponding 95% confidence intervals.

**Table 2:** Detailed primer sequences.

Primer name	Gene ID	Forward primer sequence (5’→3’)	Reverse primer sequence (5’→3’)
4E-BP1	116636	GATGGTAGATGGCTGGTGATG	GCTGCTGGTCATCTATGTTGTC
IL-4	287287	ATGGAGCTGCAGAGACTCTT	CTTAGCAGTGAGGTCTTCGT
CXCL1	81503	CTGCGCTGCTGCTGGTGATG	GTCAGAAGCCAGCGTTCACC
CXCL5	60665	GCTGCTGCTGGTGATGCTG	GGTGATGTTGAGGGAGCTG
FGF5	60662	ACAGCAACAGCAGCAGATGG	GGTGATGGTGATGGTGATGG
IL-15RA	690369	CAGCAGTGGATGGAGAAGGA	GCTGGTGAAGATGGTGAAGC
LIFR	81680	CAAGATGGTGGTGAAGGTGA	GCTGCTGGTGATGGTGATGT
PD-L1	499342	GAGTATGGCAGCAATGTCAC	CCTTTTCCCAGTACACCACT
SLAMF1	498286	ATGCTGCTGGTGATGCTGAC	GATGGTGATGGTGATGGTGC
IL-18	29197	CAGGCCTGACATCTTCTG	CTGACATGGCAGCCATT
IL-20RA	308716	GACATCAAGAAGGTGGTGAA	GAAGAATGGGAGTTGCTGTT
IL-6	24498	AAATGGGCTCCCTCTCATCAGTTC	TTGGATGGTCTTGGTCCTTAGCC
GAPDH	24383	CAACTCCCTCAAGATTGTCAGCAA	GGCATGGACTGTGGTCATGA

#### 2.2.3 Proteomic data analysis of intervertebral disc degeneration

To further explore protein-level alterations associated with intervertebral disc degeneration, publicly available proteomic data were retrieved from the PRIDE (PRoteomics IDEntifications Database, Project PXD056620). Specifically, mass spectrometry–based proteomic datasets of degenerated human intervertebral disc tissues were screened and analyzed. Protein expression profiles between degenerated and control samples were compared to identify differentially expressed proteins.

#### 2.2.4 ELISA assay

At 4 weeks after model establishment, intervertebral disc tissues were collected and processed on ice. Samples were minced and homogenized in RIPA lysis buffer (1 mL per 100 mg tissue), followed by centrifugation at 12,000 rpm for 5 min at 4 °C. The concentrations of IL-6 and IL-18 were measured using ELISA kits from Proteintech (Wuhan, China; Cat. No. KE20024) and Abcam (Cambridge, UK; Cat. No. ab213909), respectively. Absorbance values were measured at 450 nm using a Varioskan LUX multimode microplate reader (Thermo Fisher Scientific, Waltham, MA, USA), and protein levels were calculated against standard curves and normalized to the corresponding dilution multiples.

#### 2.2.5 Gut microbiota analysis (metagenomic sequencing)

To validate the findings from Mendelian randomization analysis, fecal samples were collected from control and disease groups at predefined time points and stored at -80°C until analysis. Metagenomic sequencing was performed on an Illumina platform. High-quality reads were obtained after quality control and host DNA removal. Taxonomic profiling was conducted to determine the relative abundance of microbial taxa. Comparisons between groups were performed to evaluate changes in microbial composition associated with disease conditions.

## 3 Results

### 3.1 Causal effect of gut microbiota and inflammation proteins on diseases

#### 3.1.1 Lumbar stenosis

**Figure 3:**
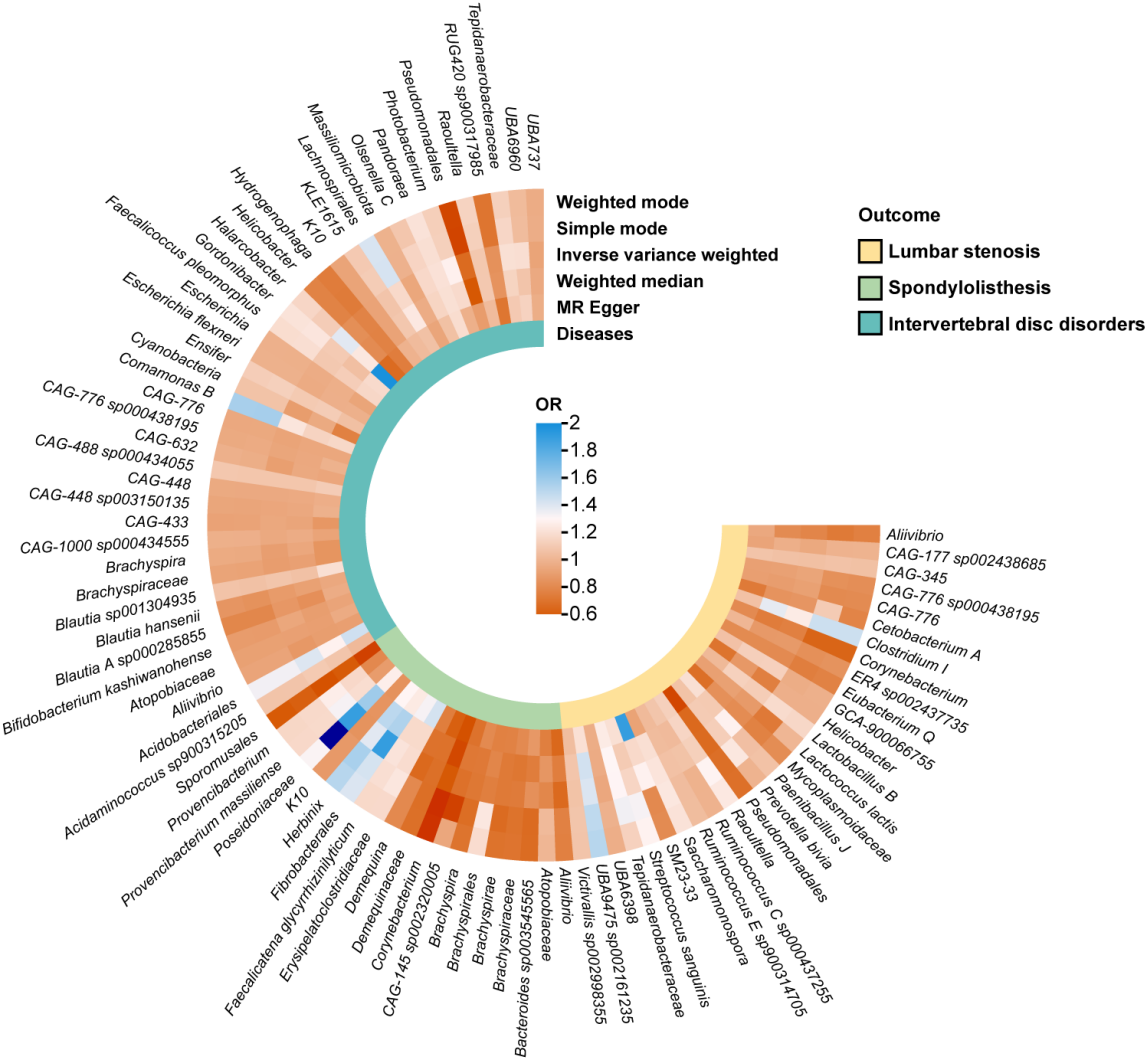
Heatmap of causal effects of gut microbial taxa on lumbar diseases. Color intensity reflects the magnitude and direction of the effect size, with warmer colors indicating increased risk (OR > 1) and cooler colors indicating protective effects (OR < 1). The analysis involved 28 bacterial taxa associated with lumbar stenosis, 20 with spondylolisthesis, and 41 with intervertebral disc disorders. Associations retained after FDR correction are summarized in the **Supplementary Table 1**. OR, odds ratio.

As shown in **Figure 3**, IVW identified 28 bacterial taxa associated with lumbar stenosis. Among these, 15 gut microbial taxa may increase the risk of lumbar stenosis. Notably, *Paenibacillus J* [OR (95%CI) = 1.280 (1.068-1.535), P = 0.007], *Tepidanaerobacteraceae* [OR (95%CI) = 1.304 (1.095-1.554), P = 0.003], and *UBA9475 sp002161235* [OR (95%CI) = 1.408 (1.179-1.682), P < 0.001] showed relatively strong risk effects. Additional taxa with significant associations are summarized in **Supplementary Table 1**.

In contrast, 13 gut microbial taxa exhibit a protective effect against lumbar stenosis. For example, *Aliivibrio* [OR (95%CI) = 0.779 (0.622-0.976), P = 0.030], *Helicobacter* [OR (95%CI) = 0.750 (0.621-0.907), P = 0.003], and *Pseudomonadales* [OR (95%CI) = 0.655 (0.451-0.952), P = 0.027] were associated with reduced risk.

**Figure 4:**
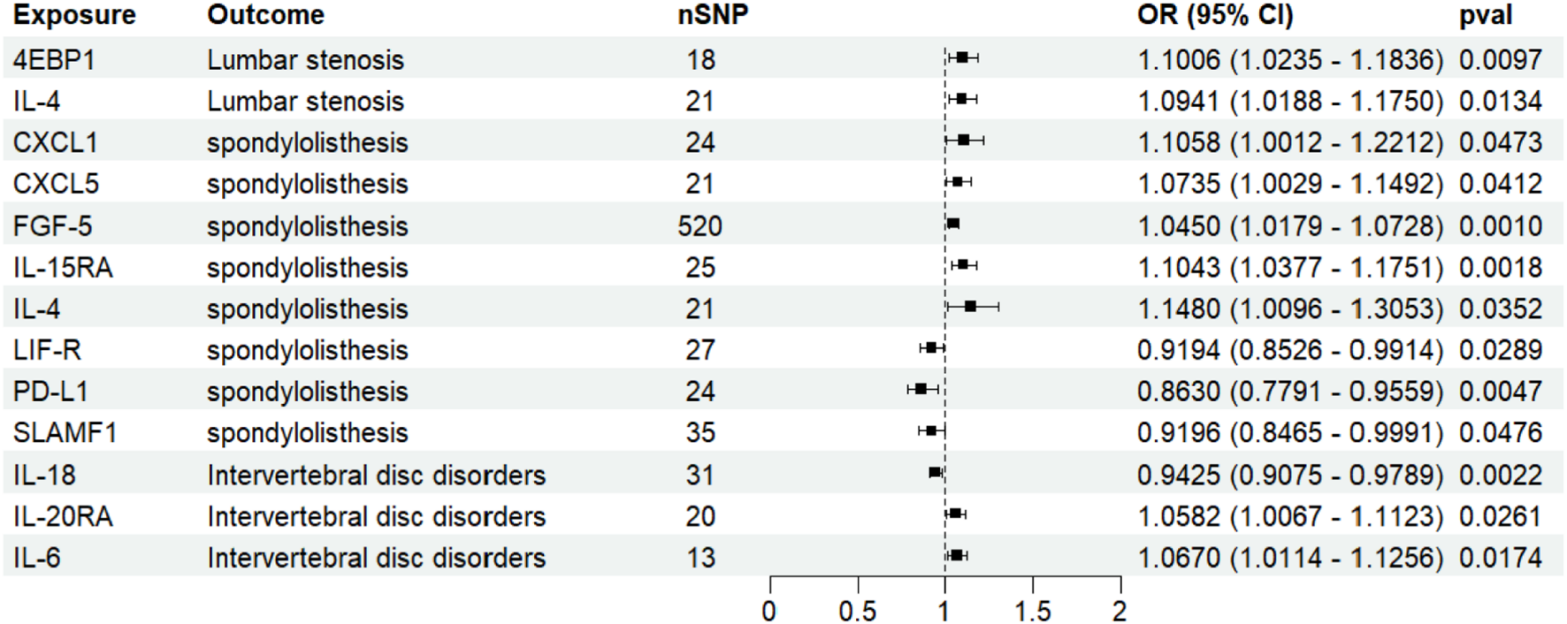
MR analysis of causal associations between inflammatory proteins and lumbar diseases. Legend: Forest plots display the causal effects of genetically predicted circulating inflammatory proteins on lumbar diseases estimated using Mendelian randomization. Associations retained after FDR correction are summarized in the **Supplementary Table 2**. OR, odds ratio; CI, confidence interval; IVW, inverse-variance weighted.

As presented in **Figure 4**, the inflammatory protein 4E-BP1 [OR (95%CI) = 1.101 (1.023-1.184), P = 0.010] and IL-4 [OR (95%CI) = 1.094 (1.019-1.175), P = 0.013] were associated with an increased risk of lumbar stenosis (**Supplementary Table 2**).

#### 3.1.2 Spondylolisthesis

As presented in **Figure 3**, IVW identified 20 bacterial taxa associated with spondylolisthesis. Seven bacterial taxa were associated with an increased risk of spondylolisthesis, including *Erysipelatoclostridiaceae* [OR (95%CI) = 1.184 (1.001-1.401), P = 0.049]; *Faecalicatena glycyrrhizinilyticum* [OR (95%CI) = 1.218 (1.029-1.441), P = 0.022]; *Fibrobacterales* [OR (95%CI) = 1.905 (1.024-3.541), P = 0.042]; *Herbinix* [OR (95%CI) = 1.386 (1.000-1.921), P = 0.049]; *Poseidoniaceae* [OR (95%CI) = 1.898 (1.209-2.979), P = 0.005]; *Provencibacterium massiliense* [OR (95%CI) = 1.327 (1.062-1.658), P = 0.013]; *Provencibacterium* [OR (95%CI) = 1.264 (1.041-1.534), P = 0.018].

On the contrary, 13 gut microbial taxa were negatively correlated with spondylolisthesis risk. Representative protective taxa included *Aliivibrio* [OR (95%CI) = 0.652 (0.476-0.893), P = 0.008]; *Corynebacterium* [OR (95%CI) = 0.572 (0.362-0.903), P = 0.017]; *Demequina* [OR (95%CI) = 0.647 (0.455-0.920), P = 0.015]; *Sporomusales* [OR (95%CI) = 0.538 (0.320-0.902), P = 0.019] (**Supplementary Table 1**).

As presented in **Figure 4**, IVW identified eight inflammatory proteins associated with spondylolisthesis. There are five inflammatory proteins that may increase the risk of spondylolisthesis. CXCL1 [OR (95%CI) = 1.106 (1.001-1.221), P = 0.047]; CXCL5 [OR (95%CI) = 1.074 (1.003-1.149), P = 0.041]; FGF-5 [OR (95%CI) = 1.045 (1.018-1.073), P = 0.001]; IL-15RA [OR (95%CI) = 1.104 (1.038-1.175), P = 0.002]; IL-4 [OR (95%CI) = 1.148 (1.010-1.305), P = 0.035]. In addition, three inflammatory proteins were associated with a decreased risk of spondylolisthesis: LIF-R [OR (95%CI) = 0.919 (0.853-0.991), P = 0.029], PD-L1 [OR (95%CI) = 0.863 (0.779-0.956), P = 0.005] and SLAMF1 [OR (95%CI) = 0.920 (0.846-0.999), P = 0.048] (**Supplementary Table 2**).

#### 3.1.3 Intervertebral disc disorders

As presented in **Figure 3**, IVW identified 41 bacterial taxa associated with other intervertebral disc disorders. There are 17 gut microbial taxa that may increase the risk of intervertebral disc disorders. Notably, *Acidobacteriales* [OR (95%CI) = 1.401 (1.052-1.866), P = 0.021], *Halarcobacter* [OR (95%CI) = 1.387 (1.078-1.783), P = 0.011], *Photobacterium* [OR (95%CI) = 1.278 (1.064-1.534), P = 0.008] showed relatively strong effects.

Conversely, 24 gut microbial taxa were negatively correlated with intervertebral disc disorders. Representative protective taxa included *Blautia A sp000285855* [OR (95%CI) = 0.863 (0.772-0.964), P = 0.009], *Hydrogenophaga* [OR (95%CI) = 0.768 (0.631-0.936), P = 0.008], *Pseudomonadales* [OR (95%CI) = 0.655 (0.501-0.857), P = 0.002], and *RUG420 sp900317985* [OR (95%CI) = 0.833 (0.702-0.989), P = 0.037] (**Supplementary Table 1**).

As presented in **Figure 4**, IL-20RA [OR (95%CI) = 1.058 (1.007-1.112), P = 0.026] and IL-6 [OR (95%CI) = 1.067 (1.011-1.126), P = 0.017] were positively associated with disease risk, whereas IL-18 [OR (95%CI) = 0.942 (0.907-0.979), P = 0.002] showed a protective effect. When FDR multiple correction was applied, the sole association that remained robust was that of IL-18 with intervertebral disc disorders (**Supplementary Table 2**).

### 3.2 Sensitivity analyses and assessment of robustness

Sensitivity analyses were conducted on all results, confirming that the associations between gut microbiota, inflammatory proteins, and the three lumbar diseases (stenosis, spondylolisthesis, and disc disorders) were robust. The scatter plots, leave-one-out plots, and funnel plots for the sensitivity analysis are shown in **Supplementary Figures 1**–**12**. To validate the results, MR Egger intercept, MR-PRESSO global test and heterogeneity tests were employed to assess horizontal pleiotropy and heterogeneity. The analysis results are presented in **Supplementary Tables 3**–**6**. The reverse MR analysis yielded no support for reverse causality.

### 3.3 Mediation analysis

**Figure 5:**
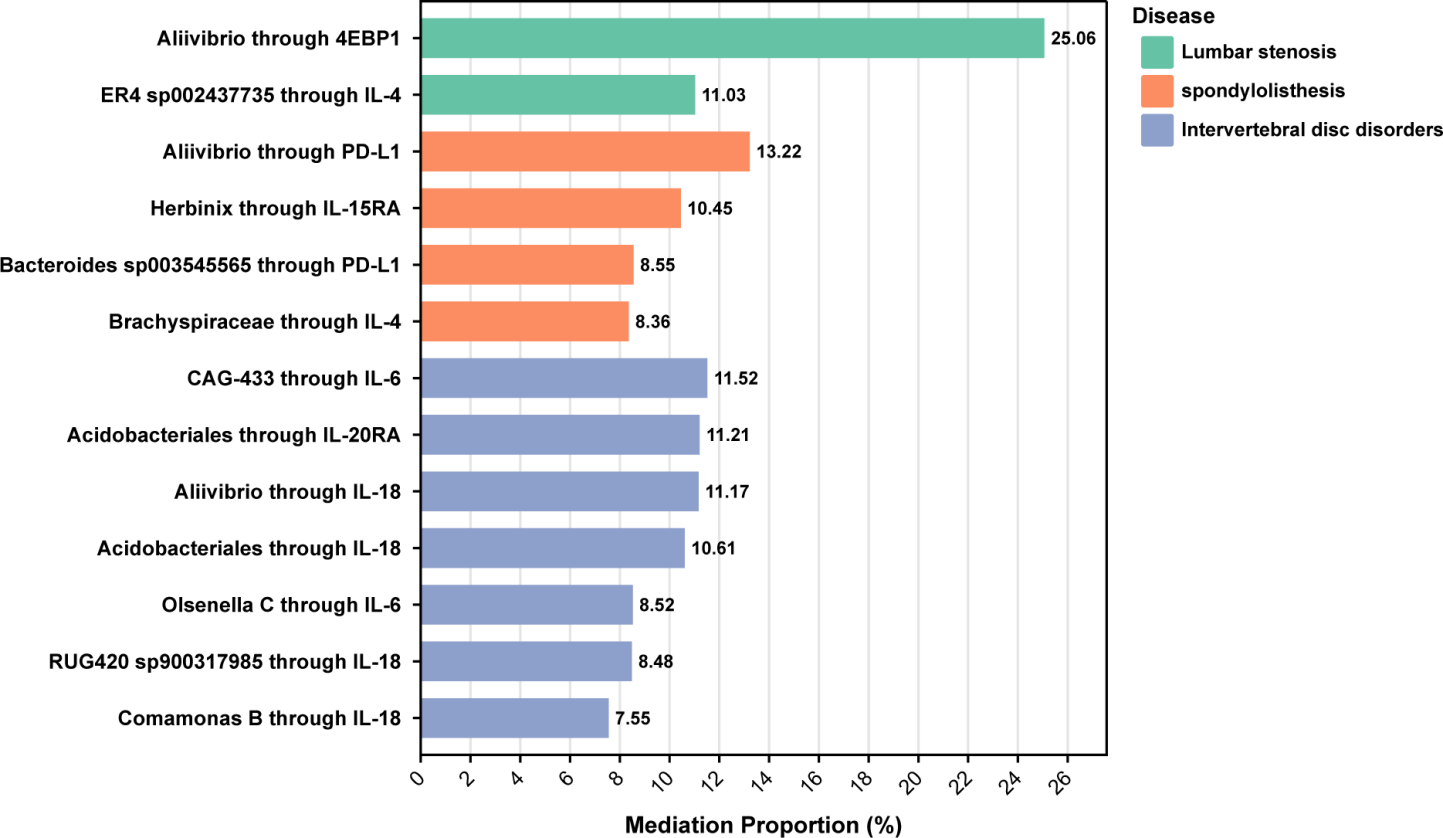
Mediation proportion of inflammatory proteins between gut microbiota and lumbar diseases. The mediation proportion represents the percentage of the total causal effect of gut microbiota on lumbar diseases that is indirectly explained through inflammatory proteins. Only mediation pathways meeting statistical significance criteria are shown.

**Figure 6:**
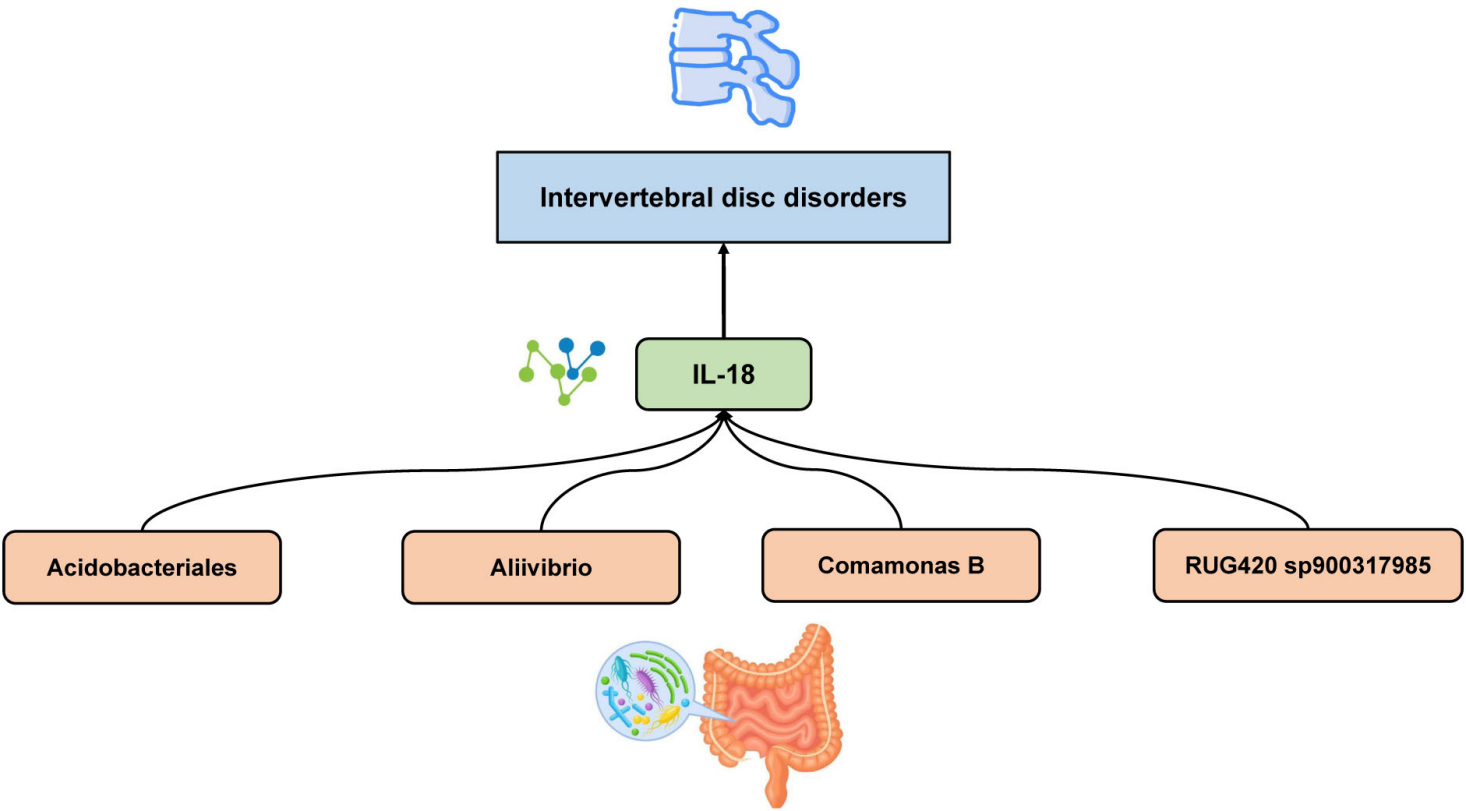
Representative mediation pathways identified by two-step MR. IL-18 mediates the effects of four bacterial taxa—*Acidobacteriales*, *Aliivibrio*, *Comamonas B*, and *RUG420 sp900317985*—on intervertebral disc disorders. This pathway suggests that modulation of gut microbiota may influence lumbar disc pathology through inflammatory signaling mediated by IL-18.

In this research, two-step MR mediation analysis was used to identify candidate pathways linking genetically predicted gut microbiota to DLSD through inflammatory proteins. By applying our screening criteria, we found 13 potential gut microbiota–inflammatory protein-lumbar diseases pathways (**Figure 5**). Notably, some inflammatory proteins mediated the effects of multiple bacterial taxa. As depicted in the **Figure 6**, PD-L1 can mediate the effects of *Aliivibrio* and *Bacteroides sp003545565* on spondylolisthesis, with mediation ratios of 13.22% and 8.55%. And both *CAG-433* and *Olsenella C* can influence the development of intervertebral disc disorders through IL-6, with mediation proportions of 11.52% and 8.52%. Furthermore, IL-18 mediated the effects of four bacterial taxa—*Acidobacteriales*, *Aliivibrio*, *Comamonas B*, and *RUG420 sp900317985*—on intervertebral disc disorders, with mediation proportions of 10.61%, 11.17%, 7.55%, and 8.48%, respectively (**Supplementary Table 7**).

### 3.4 Detection of inflammatory protein expression

To further validate the results of Mendelian randomization, we examined the mRNA expression of key inflammatory and immune-associated molecules using qPCR. In the lumbar spinal stenosis group, 4E-BP1 expression was significantly increased, while IL-4 expression was reduced compared with controls. In the spondylolisthesis group, analysis of ligamentum flavum tissues showed significant upregulation of CXCL1, CXCL5, FGF-5, LIF-R, PD-L1, and SLAMF1, whereas IL-15RA and IL-4 were markedly downregulated. In the intervertebral disc disease group, the mRNA levels of IL-6, IL-18, and IL-20RA were significantly upregulated in disc tissues compared with controls (**Figure 2E**). Overall, these results reveal heterogeneous inflammatory and immune responses across distinct lumbar degenerative disorders, characterized by prominent pro-inflammatory activation accompanied by the selective inhibition of specific immunoregulatory cytokines.

### 3.5 Proteomic data exploration and ELISA validation

**Figure 7:**
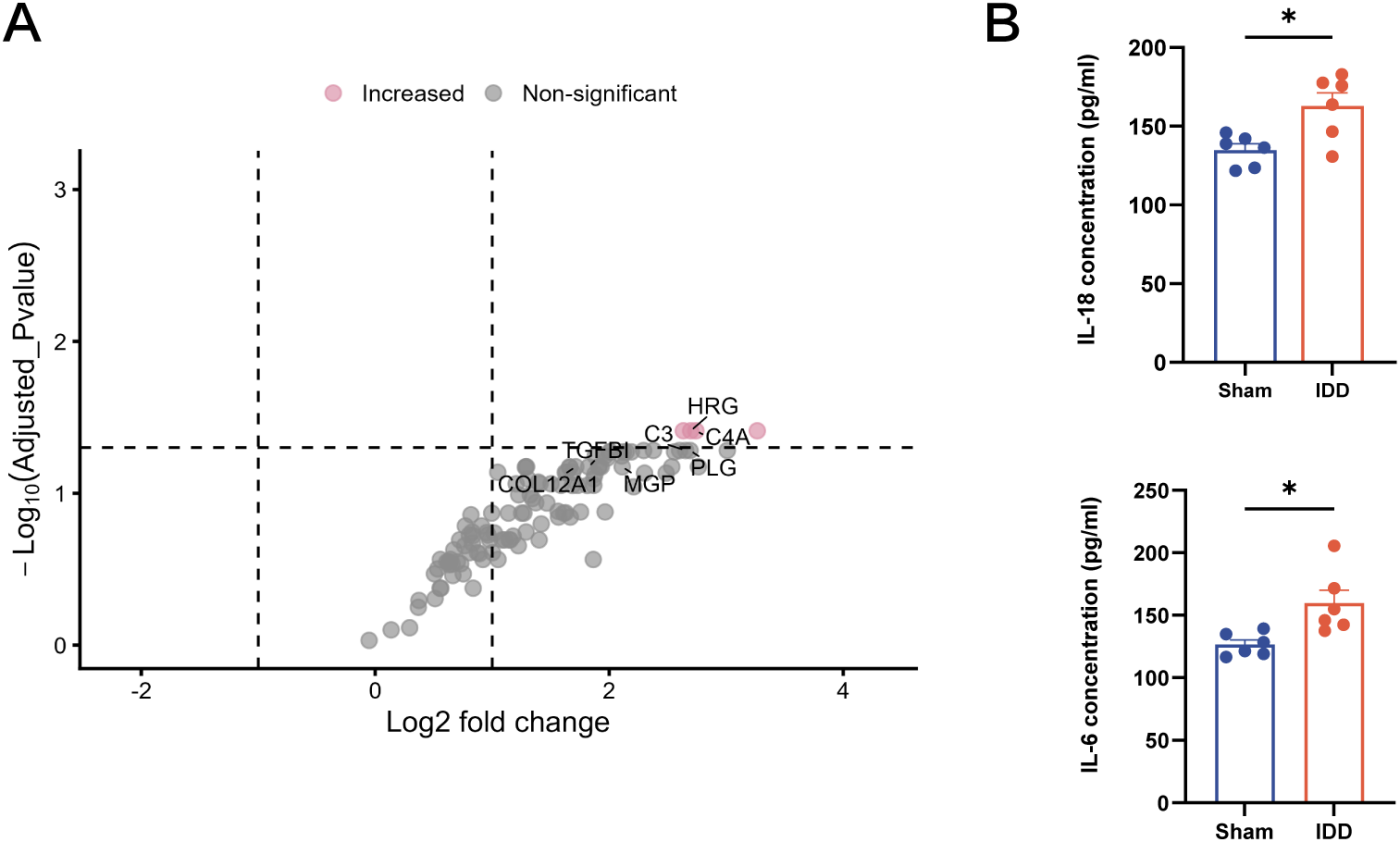
Identification and validation of pro-inflammatory markers. **(A)** Volcano plot of differentially expressed factors between Sham and IDD groups. The x-axis represents log_2_ fold change, and the y-axis shows -log_10_ adjusted P-value. Dashed lines indicate thresholds for significance (|log_2_FC| > 1 and adjusted P < 0.05). Upregulated factors are highlighted in pink, while non-significant factors are shown in gray. Selected upregulated proteins, including HRG and C4A, are labeled. **(B)** ELISA validation of inflammatory cytokines. Concentrations of IL-18 and IL-6 were measured in Sham and IDD groups. Both cytokines were significantly increased in the IDD group. Data are presented as mean ± SEM (n = 6 per group). (*p < 0.05, one-way ANOVA on ranks post Dunn’s Method).

Analysis of proteomic datasets from the PRIDE database did not reveal overlapping proteins with those identified by Mendelian randomization. This lack of concordance may reflect differences in sample source, disease stage, tissue specificity, analytical platform, and the distinction between tissue proteomics and genetically proxied circulating protein liability (**Figure 7A**). However, the ELISA results demonstrated that IL-6 and IL-18 were significantly increased in the model group compared with controls (**Figure 7B**). These findings provide context-specific support for the involvement of selected inflammatory proteins in intervertebral disc degeneration despite the lack of concordance with publicly available proteomic data.

### 3.6 Gut microbiota alterations based on metagenomic sequencing

**Figure 8:**
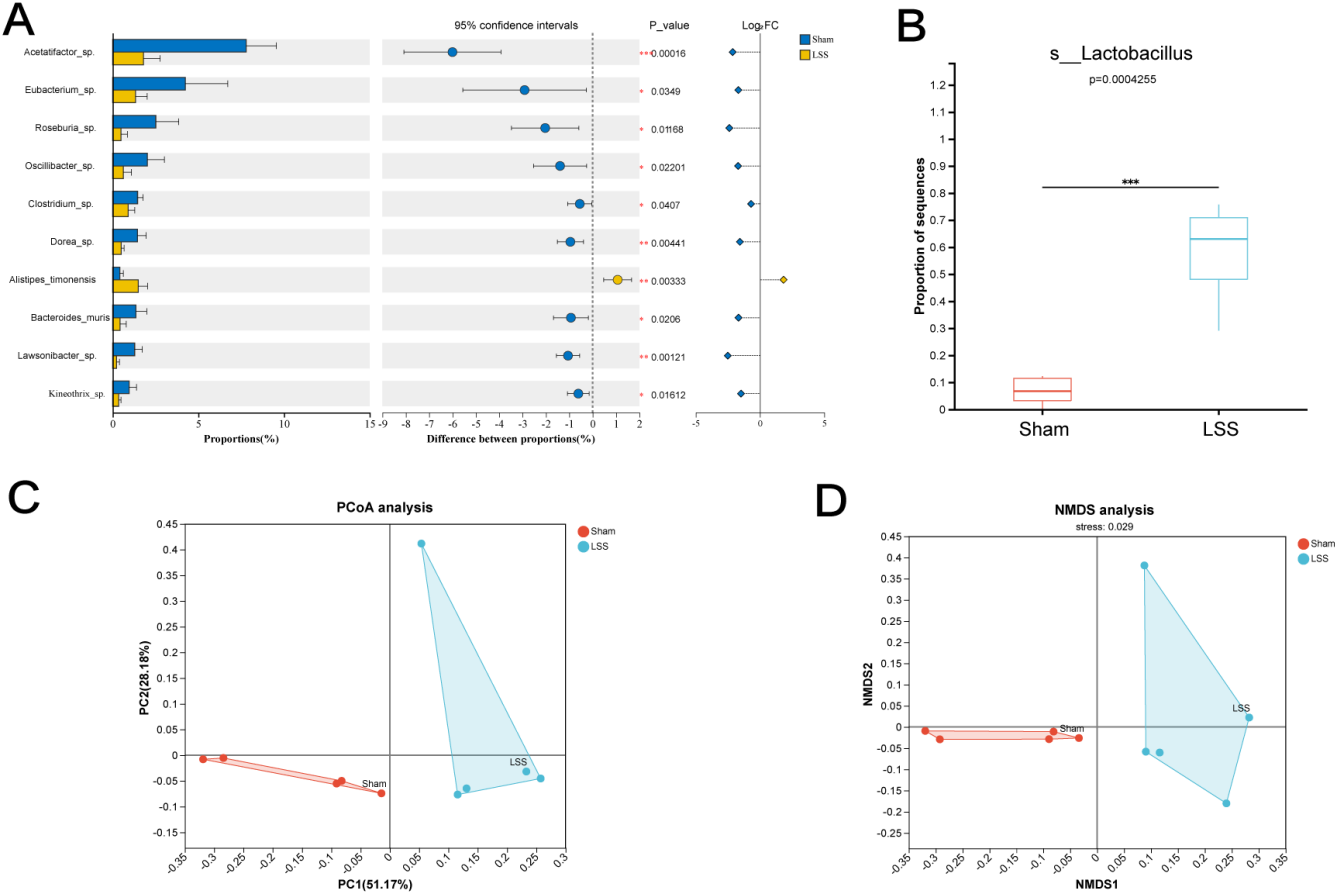
Gut microbiota diversity and differential species in LSS model rats. **(A)** Relative abundance of significantly differential bacterial species between groups. (*P<0.05, **P<0.01, ***P<0.001) **(B)** Comparison of Lactobacillus Abundance. Box plot representing the proportion of sequences assigned to the genus Lactobacillus in the Sham and LSS groups. A marked increase in the relative abundance of Lactobacillus is observed in the LSS group. **(C)** Principal Coordinates Analysis (PCoA). Distinct clustering of the Sham (red) and LSS (blue) groups indicates a significant shift in microbial community structure. **(D)** Non-metric Multidimensional Scaling (NMDS). NMDS plot further confirming the separation of microbial profiles between experimental groups.

Metagenomic analysis revealed only partial consistency between gut microbiota alterations and Mendelian randomization findings, and this concordance was mainly observed in the lumbar spinal stenosis group. Specifically, the relative abundance of Eubacterium and Lactobacillus was significantly altered in the disease group compared with controls (p < 0.05), suggesting that some genetically prioritized microbial signals may be reflected in the experimental model (**Figure 8**). However, no significant differences were observed for most MR-identified microbial taxa in the spondylolisthesis or intervertebral disc disease groups. This limited overlap likely reflects differences between human genetically proxied microbial liability and the short-term, species-specific experimental conditions of the rat models.

## 4 Discussion

In this two-sample Mendelian randomization study, we integrated genetic instruments for gut microbial taxa, circulating inflammatory proteins, and three major degenerative lumbar spine disorders to evaluate both direct associations and potential mediation pathways. Several features of the results deserve emphasis. First, genetically proxied gut microbiota showed significant associations with all three lumbar phenotypes, supporting a non-random link between microbial liability and lumbar degeneration. Second, the associations of inflammatory proteins varied across the three outcomes. This suggests that some immune signals are shared, while others are enriched in specific phenotypes. Third, we identified 13 candidate pathways linking gut microbiota to lumbar degeneration via inflammatory proteins. These mediation results indicate that inflammatory proteins may partly carry the effects of gut microbiota on disease development, supporting a microbiota-inflammation-degeneration framework rather than isolated pairwise relationships.

The ORs reported in this study should be interpreted as genetically proxied relative risk estimates rather than direct clinical effect sizes. Most of the significant associations were modest in magnitude, suggesting susceptibility signals that are biologically informative but not necessarily large enough to function as standalone clinical markers. Likewise, the mediation proportions of 7.55%-13.22% indicate small-to-moderate indirect contributions to the total effect, which are meaningful in a multifactorial disease context but do not imply that inflammatory proteins explain most of the microbiota-related risk.

An important implication of the present study is that intervertebral disc disorders, degenerative spondylolisthesis, and lumbar spinal stenosis may share upstream biological susceptibility despite their different dominant tissue manifestations. Intervertebral disc disorders are mainly characterized by disc matrix degradation, cellular senescence, and pain-related inflammatory sensitization (11, 12). Lumbar spinal stenosis is more closely linked to hypertrophy and fibrotic remodeling of the ligamentum flavum, facet-related degeneration, and progressive canal narrowing (15, 16). Degenerative spondylolisthesis reflects a broader functional spinal unit disorder in which disc degeneration, facet arthropathy, ligamentous degeneration, and instability interact over time (7, 42). However, all three phenotypes appear to converge on chronic inflammatory remodeling, altered extracellular matrix homeostasis, and persistent mechanical stress. Our findings on gut microbiota reveal both shared patterns and distinct features across lumbar degeneration. The observation that each condition had significant taxon associations challenges the view that microbiota−related genetic risk applies to only one lumbar phenotype. This pattern is consistent with prior Mendelian randomization studies linking gut microbial variation to intervertebral disc degeneration, back pain, and lumbar spinal stenosis (20–22, 43). However, the number, direction, and distribution of associated taxa were not identical across the three outcomes. This argues against a simple one-taxon-one-disease interpretation. Instead, it suggests that the gut microbiota may influence a broader background of inflammatory or metabolic susceptibility. Recent studies in spinal cord injury have similarly shown injury-induced noncoding RNA dysregulation and fibrotic remodeling, indirectly supporting the plausibility of shared inflammatory and repair-related responses across spinal disorders (44–47).

The lumbar spinal stenosis results merit specific consideration. We identified 28 associated gut microbial taxa and observed positive associations for 4E-BP1 and interleukin-4. The inflammatory interpretation of stenosis is biologically plausible because ligamentum flavum hypertrophy is increasingly recognized as an active inflammatory and profibrotic process rather than a purely passive manifestation of ageing (15, 16). 4E-BP1 is a downstream effector in translational control and has been implicated in fibrotic signaling and tissue remodeling in other organ systems (48, 49). Although interleukin-4 is classically viewed as anti-inflammatory in some contexts, cytokine signaling in chronic fibroproliferative disease often depends on the context. The stenosis findings therefore suggest that microbiota-related immune perturbation may converge on translational and cytokine pathways that favor canal-narrowing tissue remodeling.

The spondylolisthesis results point to a broader and more immunologically diverse inflammatory profile. Degenerative spondylolisthesis had a broader set of positively associated inflammatory proteins than lumbar spinal stenosis or intervertebral disc disorders. These included CXCL1, CXCL5, FGF−5, IL−15RA, and interleukin−4. In contrast, LIF−R, PD−L1, and SLAMF1 appeared protective. This is noteworthy because degenerative spondylolisthesis is not merely vertebral slippage; it reflects coordinated degeneration of the disc, facet joint, ligamentous structures, and segmental stabilizers (7, 42). Chemokines such as CXCL1 and CXCL5 are biologically plausible in this setting because they participate in leukocyte recruitment, inflammatory amplification, and pain-related signaling (50, 51). PD-L1 is particularly intriguing because it emerged both as a protein associated with lower spondylolisthesis risk and as a mediator of microbiota–disease pathways. Experimental studies have shown that PD-L1 signaling can suppress nociception and modulate immune homeostasis (52), while related immune checkpoint biology has been implicated in osteoarthritic synovitis and joint inflammation (53). These results suggest that degenerative spondylolisthesis may be especially informative for understanding how microbiota-related immune variation intersects with instability, facet degeneration, and joint-like inflammatory remodeling.

The intervertebral disc disorder results further strengthen the inflammatory interpretation of lumbar degeneration. We identified 41 associated microbial taxa and observed positive associations for IL-20RA and IL-6, whereas IL-18 showed an inverse association that remained robust after false-discovery-rate correction. Disc degeneration is widely recognized as an inflammatory and catabolic process in which cytokines drive extracellular matrix breakdown, cell stress, and pain generation (11, 12). IL-6 has repeatedly been linked to disc degeneration severity and symptom-related biology (13, 14), making its appearance in our results biologically coherent. IL-18 is more complex. Experimental work has linked IL-18 signaling to catabolic responses in disc cells (54), and inhibition of IL-18 can attenuate disc degeneration in model systems (55). The inverse direction observed here may reflect the distinction between genetically proxied circulating levels and local tissue activity, or it may indicate compensatory immune regulation. Nonetheless, because IL-18 remained robust after multiple-testing correction and mediated several microbiota–IDD associations, it should be regarded as an important candidate for further validation.

A key scientific contribution of this work is that we directly assessed whether inflammatory proteins act as mediators between gut microbiota and lumbar degeneration. Earlier studies often looked at gut microbiota–disease links or inflammatory protein–disease links in isolation. However, few have formally examined if inflammatory proteins might sit in an intermediate layer connecting microbial variation to spinal outcomes (25, 30). Our mediation analyses identified 13 candidate gut microbiota–inflammatory protein–disease pathways. This is important for two reasons. First, it supports the view that inflammation is not merely a parallel correlate of lumbar degeneration but may constitute a measurable intermediate mechanism through which microbiota-related effects are transmitted (11, 12). Second, the topology of mediation was clearly many-to-many, with multiple taxa converging on shared inflammatory nodes and some inflammatory proteins mediating more than one microbial association. Such a structure is biologically more plausible for chronic multifactorial disease than a single-cascade model and closely resembles the complex mediation architecture described in other microbiota–inflammation studies (8, 10). The mediation findings also clarify what is shared and what is distinct across the three lumbar phenotypes. We did not identify a single universal microbial or inflammatory signature present across all diseases. Instead, we observed partial overlap at the level of inflammatory logic and specific mediator reuse. PD-L1-mediated effects were evident in spondylolisthesis, whereas IL-6 and IL-18 mediated multiple microbiota associations with intervertebral disc disorders. In lumbar spinal stenosis, 4E-BP1 mediated several taxa–disease links. This suggests that the commonality across IDD, DS, and LSS may lie less in a fixed biomarker set and more in a shared principle: gut microbiota-associated liability appears to be translated into disease risk through inflammatory or remodeling-related intermediates, but the exact proteins involved may vary according to tissue compartment, biomechanical burden, and degenerative stage. That distinction is important because it justifies a unified analytical framework even when the final mediator sets are not identical.

Methodologically, the findings were supported by multiple robustness checks. Our main analysis relied on inverse−variance weighted MR, supported by weighted median, weighted mode, simple mode, and MR−Egger (28, 36). To evaluate heterogeneity and horizontal pleiotropy, we applied Cochran’s Q, the MR−Egger intercept, and MR−PRESSO (37). Reverse MR analyses did not support disease-driven effects on the identified exposures, which reduces but does not eliminate concerns about reverse causation. Collectively, these checks strengthen confidence that the observed associations are not simply artifacts of residual confounding, reverse directionality, or single-instrument influence.

From a translational perspective, the results are relevant at several levels. First, circulating inflammatory proteins supported by genetic evidence may provide a more focused set of biomarker candidates than proteins identified only in cross-sectional association studies (33). Second, by placing inflammatory proteins between gut microbiota and disease, the study narrows the gap between distal microbial exposures and clinically actionable molecular pathways (25, 33). Third, if future studies validate shared and phenotype-specific inflammatory nodes, microbiota-informed immune profiling may eventually complement imaging and symptom-based classification, helping to distinguish predominantly fibrotic-stenotic, instability-inflammatory, or disc-catabolic molecular patterns. Finally, the identification of mediators with partial but measurable effects provides a more realistic translational framework than a deterministic biomarker model, because it better reflects the multifactorial nature of lumbar degeneration.

Several limitations should be considered. The exposure and outcome data were primarily derived from European populations, which may limit generalizability. And the complete individual-level overlap between the microbiota GWAS and the FinnGen outcome datasets could not be fully verified. In addition, microbial instruments reflect taxonomic abundance rather than functional activity, and circulating inflammatory markers may not fully represent local tissue conditions. Notably, proteomic data mining from publicly available datasets (e.g., PRIDE) did not reveal consistent overlap with MR-identified inflammatory proteins. This discrepancy may be attributed to heterogeneity in human samples, differences in disease stage, and the limited sensitivity of mass spectrometry for detecting low-abundance or membrane-associated proteins. Furthermore, the qPCR results were not entirely consistent with MR findings, likely due to differences between long-term genetic effects and tissue-specific expression under pathological conditions (31). Furthermore, ELISA validation was conducted only in intervertebral disc tissues, and all experimental validation was performed in animal models, which may limit direct clinical translation. Future studies incorporating human tissue or clinical samples will be necessary to confirm the relevance of these findings in patients. As a membrane-bound receptor, IL-20RA is not typically secreted into extracellular fluids, making it less suitable for conventional ELISA-based detection. Despite these limitations, the overall trend of inflammatory activation was broadly consistent across MR and experimental analyses, supporting the robustness of the observed associations. Several signals identified in this study, including IL-18, PD-L1, and 4E-BP1, require cautious interpretation. Although implicated by MR and mediation analyses, they should not be considered confirmed mechanistic drivers. MR reflects lifelong genetically proxied circulating protein effects, whereas qPCR and ELISA capture local tissue-level expression at specific disease stages, and these layers do not necessarily align. In addition, these proteins have context-dependent and pleiotropic roles in immune regulation. Finally, MR-based mediation identifies candidate pathways but does not establish classical biological mediation. Therefore, these signals should be interpreted as hypothesis-generating rather than definitive mechanistic evidence.

In addition, the gut microbiota findings should be interpreted with caution. Only a subset of microbial taxa identified by Mendelian randomization were detectable or significantly altered in the experimental models, while many taxa showed low abundance or were not detected. This discrepancy may reflect fundamental differences between human microbiome-based genetic associations and animal-based microbiota profiling. MR analyses capture lifelong host genetic influences on microbial composition in large human populations, whereas animal experiments represent controlled and relatively short-term perturbations under specific environmental and dietary conditions. Furthermore, interspecies differences in gut microbiota composition between humans and rodents are well recognized, with certain taxa being human-specific or present at very low abundance in rodents. Technical factors, including sequencing depth, taxonomic resolution, and the use of reference databases, may also contribute to the limited detection of specific taxa. Importantly, the partial overlap observed in the lumbar spinal stenosis model suggests that certain microbiota–disease associations may be more robust or conserved across species, whereas others may be context-dependent. Therefore, the absence of detectable changes in some taxa does not necessarily contradict the MR findings but rather highlights the complexity of translating population-level associations into experimental systems. Future studies using human cohorts, longitudinal sampling, and functional microbiome analyses will be essential to further validate these observations.

The present study supports a model in which gut microbiota and inflammatory proteins form an interconnected network contributing to degenerative lumbar spine disorders. The findings highlight both shared inflammatory mechanisms and phenotype-specific pathways, and provide a framework for future translational studies targeting microbiota–immune interactions in lumbar degeneration.

## 5 Conclusions

Using a unified two-sample MR framework across gut microbiota, circulating inflammatory proteins, and three degenerative lumbar spine disorders, we identified genetically supported microbiota associations with disease risk and demonstrated that inflammatory proteins may partially mediate key microbiota–disease relationships. These findings were further supported by experimental validation, including qPCR, ELISA, and gut microbiota sequencing, which revealed consistent inflammatory activation and microbial alterations in lumbar tissues. These results prioritize immune mediators as actionable candidates for mechanistic validation and translational development in degenerative lumbar spine disorders.

## Data Availability

The original contributions presented in the study are included in the article/**Supplementary Material**. Further inquiries can be directed to the corresponding authors.

## Glossary

IDD: intervertebral disc disorders
DLSD: Degenerative lumbar spine disorders
DS: degenerative spondylolisthesis
LSS: lumbar spinal stenosis
LBP: low back pain
GM: gut microbiota
MR: Mendelian randomization
IPs: inflammatory proteins
FDR: false discovery rate
4E-BP1: eukaryotic translation initiation factor 4E-binding protein 1
IL-4: interleukin-4
CXCL1: C-X-C motif chemokine ligand 1
CXCL5: C-X-C motif chemokine ligand 5
FGF-5: fibroblast growth factor 5
IL-15RA: interleukin-15 receptor alpha
IL-20RA: interleukin-20 receptor subunit alpha
IL-6: interleukin-6
IL-18: interleukin-18
PD-L1: programmed death-ligand 1
DS: degenerative spondylolisthesis
GWAS: genome-wide association study
SNP: single nucleotide polymorphism
pQTL: protein quantitative trait locus
NPX: Normalized Protein eXpression
SD: standard deviation
IV: instrumental variable
LD: linkage disequilibrium
IVW: inverse variance weighted
MR-Egger: Mendelian randomization Egger regression
MR-PRESSO: Mendelian Randomization Pleiotropy RESidual Sum and Outlier
LIF-R: leukemia inhibitory factor receptor
SLAMF1: signaling lymphocytic activation molecule family member 1
OR: odds ratio
CI: confidence interval
STROBE-MR: Strengthening the Reporting of Observational Studies in Epidemiology using Mendelian Randomization
qPCR: Quantitative Polymerase Chain Reaction
PRIDE: PRoteomics IDEntifications Database
ELISA: Enzyme-Linked Immunosorbent Assay
