# Supplementary material for: Inflammatory protein mediators linking gut microbiota to degenerative lumbar spine disorders: cross-disease genetic evidence
Source: Front Immunol. 2026 Jun 3;17:1855966. doi: 10.3389/fimmu.2026.1855966 (PMC13272031; doi:10.3389/fimmu.2026.1855966)
Supplement: Supplementary file 1 [file Table1.docx]

**Supplementary information**

**Figure S1**: Scatter plot for gut microbiotas on lumbar stenosis.

**Figure S2**: Scatter plot for gut microbiotas on spondylolisthesis.

**Figure S3**: Scatter plot for gut microbiotas on intervertebral disc disorders.

**Figure S4**: Funnel plot for gut microbiotas on lumbar stenosis.

**Figure S5**: Funnel plot for gut microbiotas on spondylolisthesis.

**Figure S6**: Funnel plot for gut microbiotas on intervertebral disc disorders.

**Figure S7**: Leave-one-out analysis for gut microbiotas on lumbar stenosis.

**Figure S8**: Leave-one-out analysis for gut microbiotas on spondylolisthesis.

**Figure S9**: Leave-one-out analysis for gut microbiotas on intervertebral disc disorders.

**Figure S10**: Scatter plot for inflammatory proteins on lumbar diseases.

**Figure S11**: Funnel plot for inflammatory proteins on lumbar diseases.

**Figure S12**: Leave-one-out analysis for inflammatory proteins on lumbar diseases.

**Figure S13:** Gut microbiota sequencing analysis in rat models of lumbar spondylolisthesis and disc herniation


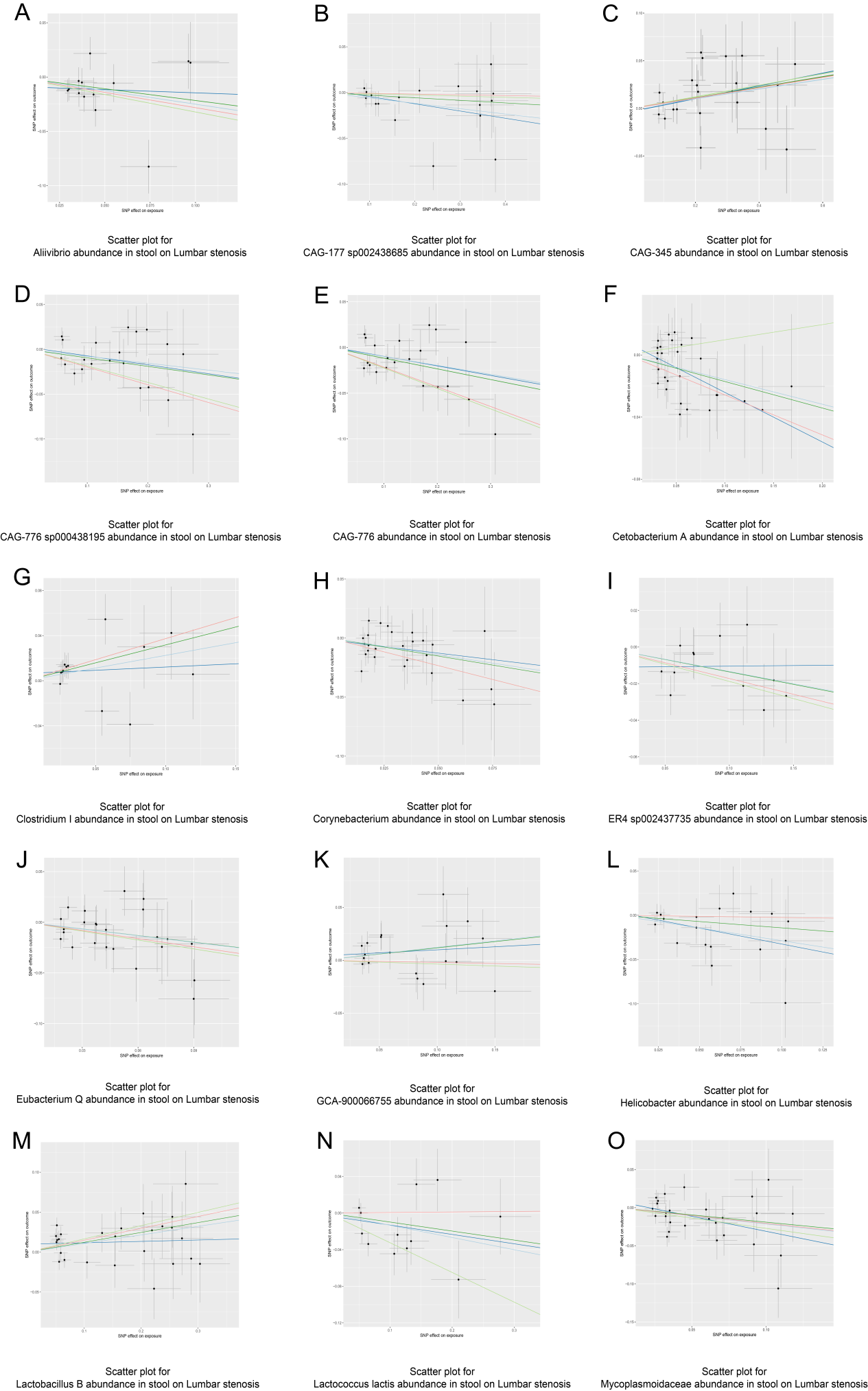


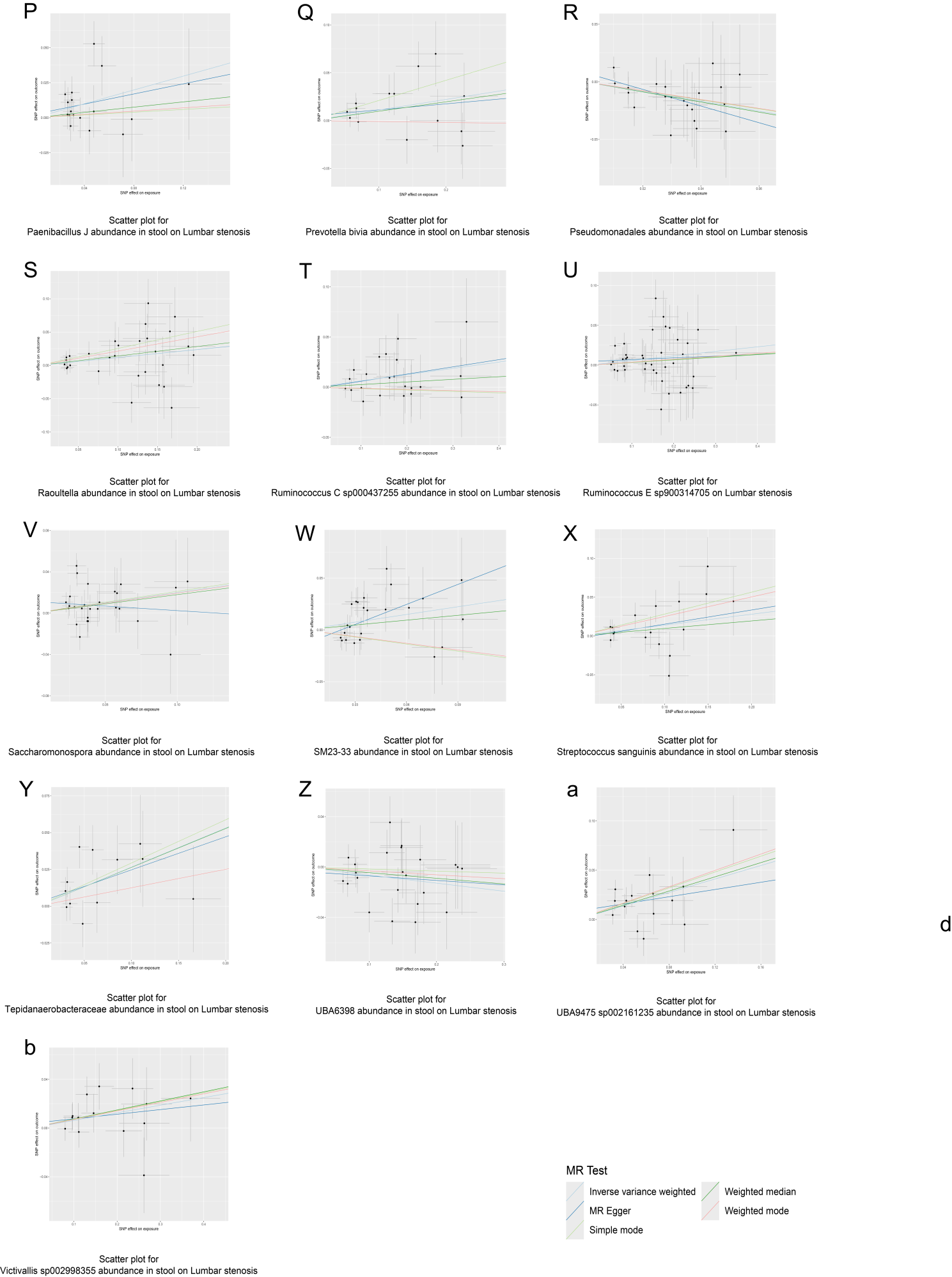


**Figure S1.** Scatter plot for gut microbiotas on lumbar stenosis. The analyses of (A) *Aliivibrio* abundance in stool (B) *CAG-177 sp002438685* abundance in stool (C) *CAG-345* abundance in stool (D) *CAG-776 sp000438195* abundance in stool (E) *CAG-776* abundance in stool (F) *Cetobacterium A* abundance in stool (G) *Clostridium I* abundance in stool (H) *Corynebacterium* abundance in stool (I) *ER4 sp002437735* abundance in stool (J) *Eubacterium Q* abundance in stool (K) *GCA-900066755* abundance in stool (L) *Helicobacter* abundance in stool (M) *Lactobacillus B* abundance in stool (N) *Lactococcus lactis* abundance in stool (O) *Mycoplasmoidaceae* abundance in stool (P) *Paenibacillus J* abundance in stool (Q) *Prevotella bivia* abundance in stool (R) *Pseudomonadales* abundance in stool (S) *Raoultella* abundance in stool (T) *Ruminococcus C sp000437255* abundance in stool (U) *Ruminococcus E sp900314705* abundance in stool (V) *Saccharomonospora* abundance in stool (W) *SM23-33* abundance in stool (X) *Streptococcus sanguinis* abundance in stool (Y) *Tepidanaerobacteraceae* abundance in stool (Z) *UBA6398* abundance in stool (a) *UBA9475 sp002161235* abundance in stool (a) *Victivallis sp002998355* abundance in stool.

.


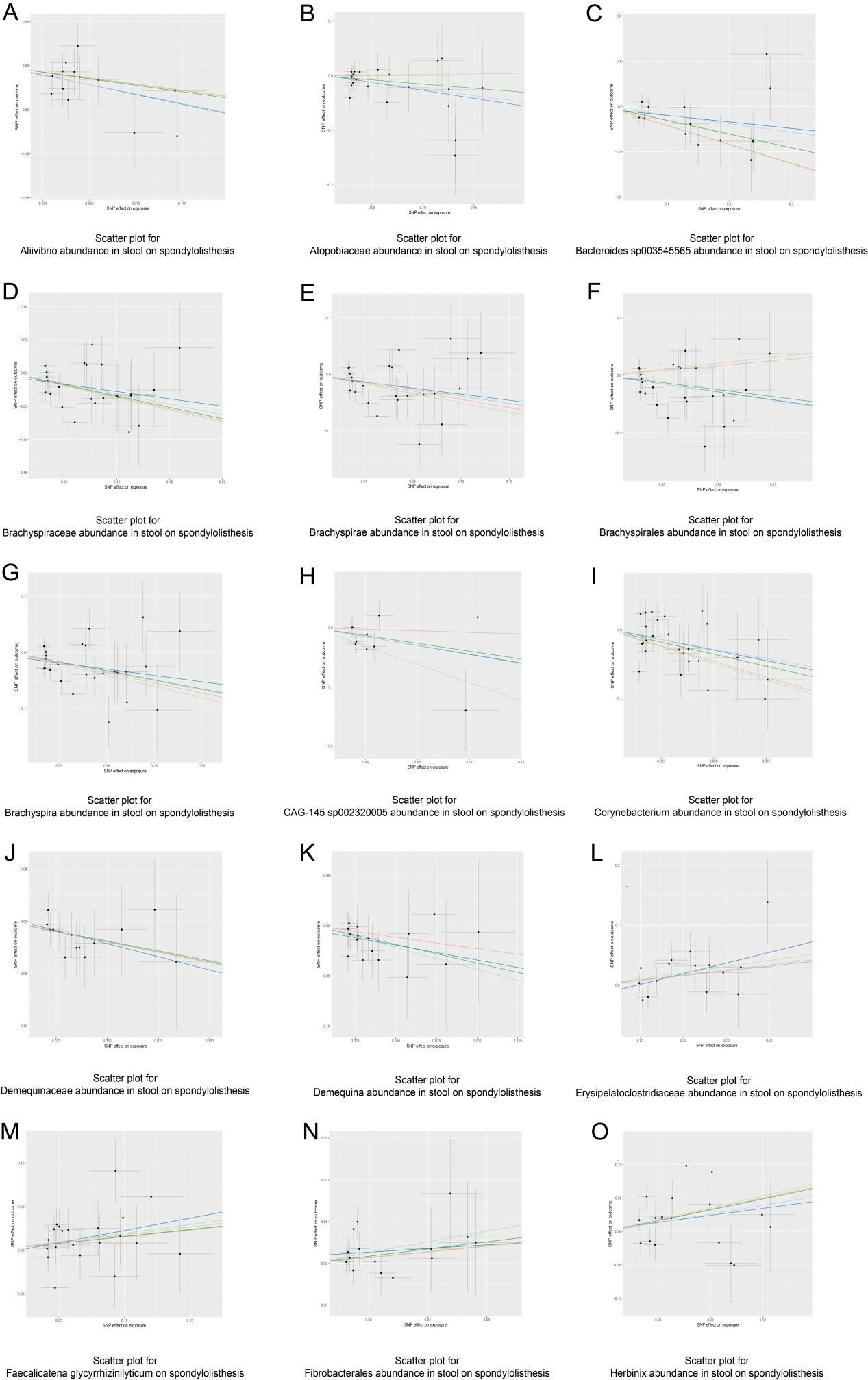


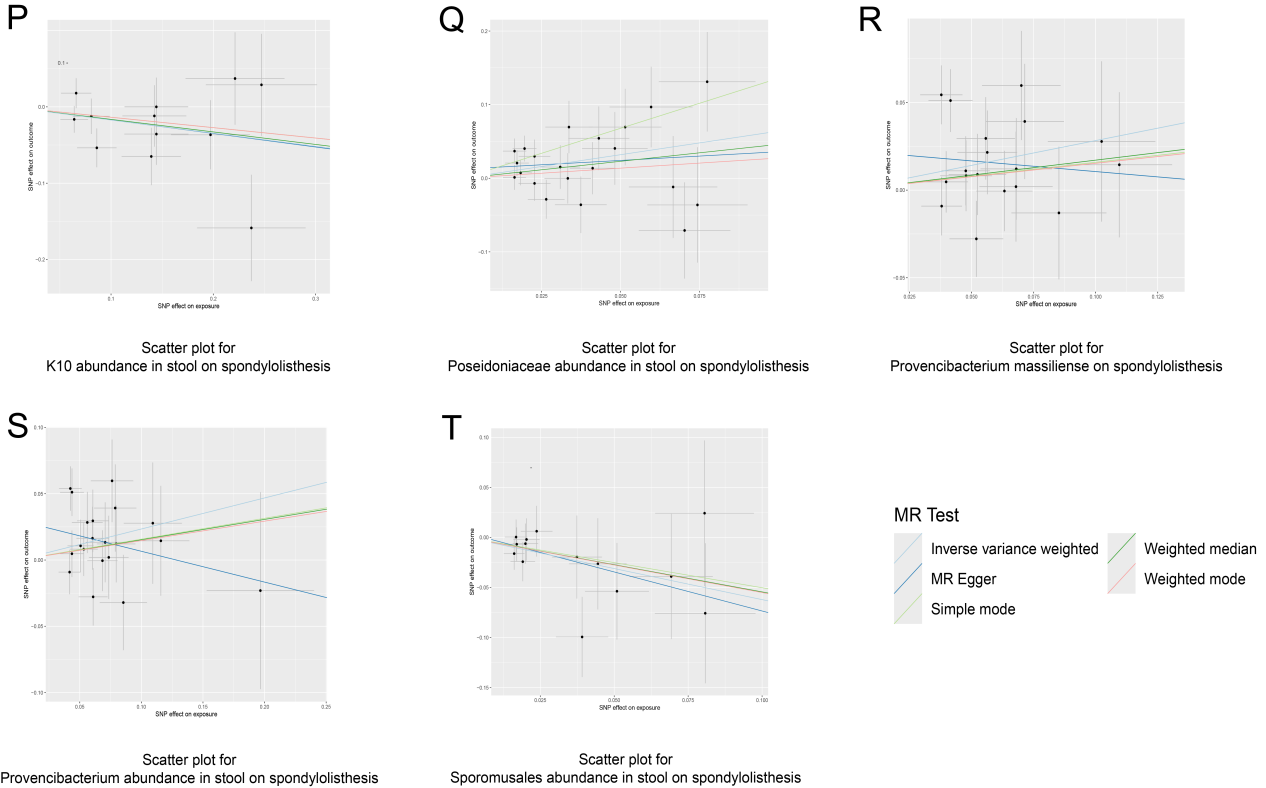


**Figure S2.** Scatter plot for gut microbiotas on spondylolisthesis. The analyses of (A) *Aliivibrio* abundance in stool (B) *Atopobiaceae* abundance in stool (C) *Bacteroides sp003545565* abundance in stool (D) *Brachyspiraceae* abundance in stool (E) *Brachyspirae* abundance in stool (F) *Brachyspirales* abundance in stool (G) *Brachyspira* abundance in stool (H) *CAG-145 sp002320005* abundance in stool (I) *Corynebacterium* abundance in stool (J) *Demequinaceae* abundance in stool (K) *Demequina* abundance in stool (L) *Erysipelatoclostridiaceae* abundance in stool (M) *Faecalicatena glycyrrhizinilyticum* abundance in stool (N) *Fibrobacterales* abundance in stool (O) *Herbinix* abundance in stool (P) *K10* abundance in stool (Q) *Poseidoniaceae* abundance in stool (R) *Provencibacterium massiliense* abundance in stool (S) *Provencibacterium* abundance in stool (T) *Sporomusales* abundance in stool.

.


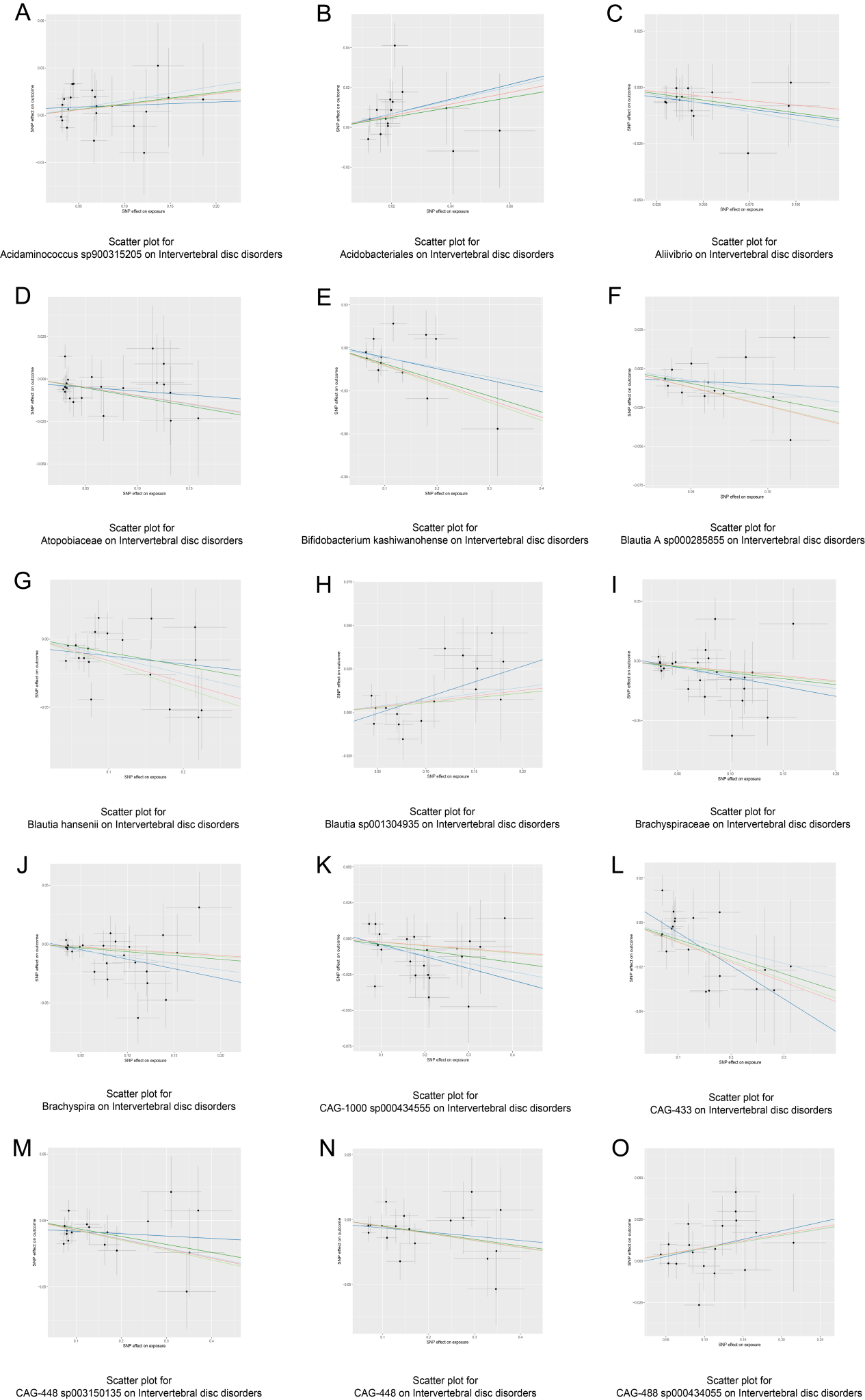


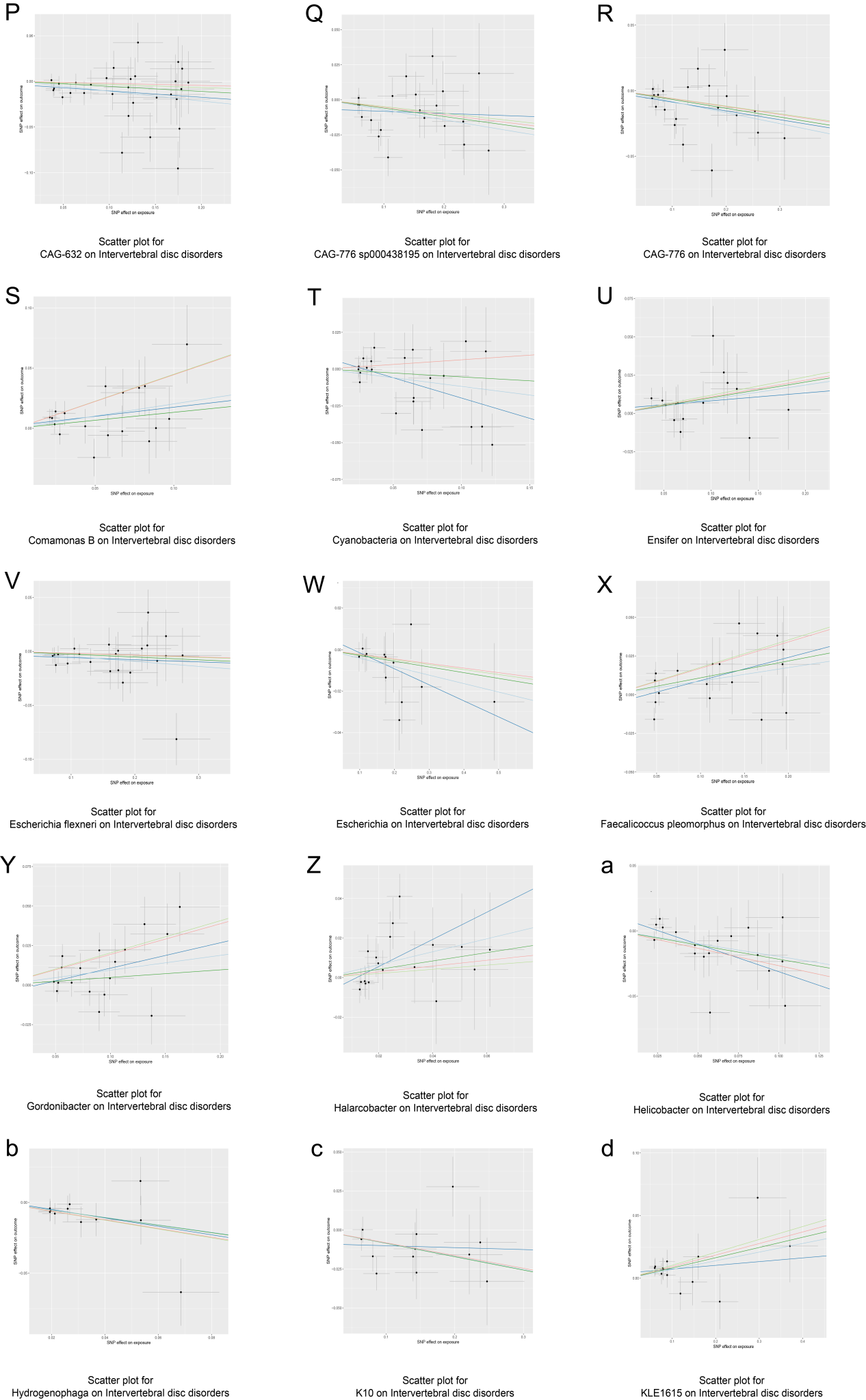


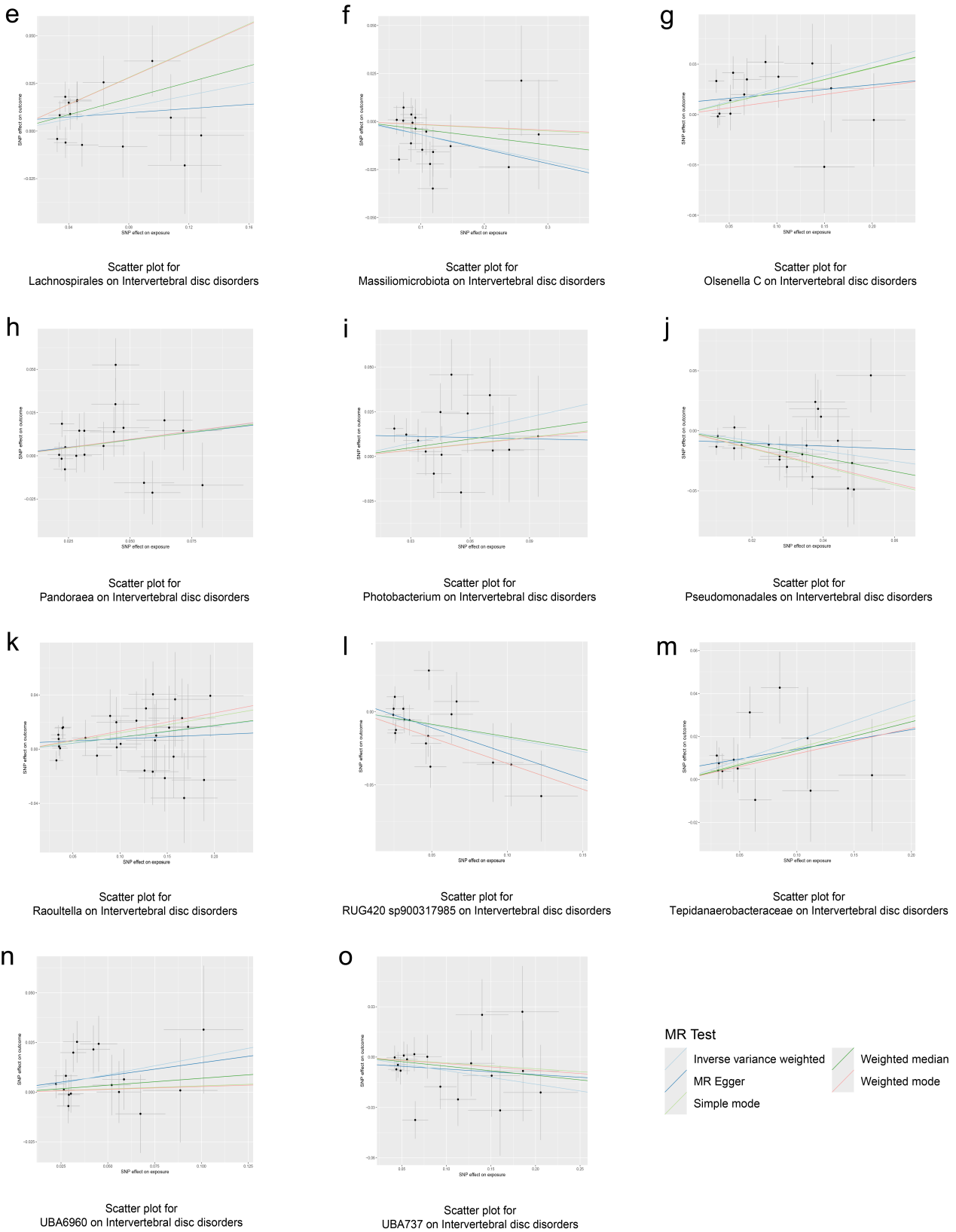


**Figure S3.** Scatter plot for gut microbiotas on intervertebral disc disorders. The analyses of (A) *Acidaminococcus sp900315205* abundance in stool (B) *Acidobacteriales* abundance in stool (C) *Aliivibrio* abundance in stool (D) *Atopobiaceae* abundance in stool (E) *Bifidobacterium kashiwanohense* abundance in stool (F) *Blautia A sp000285855* abundance in stool (G) *Blautia hansenii* abundance in stool (H) *Blautia sp001304935* abundance in stool (I) *Brachyspiraceae* abundance in stool (J) *Brachyspira* abundance in stool (K) *CAG-1000 sp000434555* abundance in stool (L) *CAG-433* abundance in stool (M) *CAG-448 sp003150135* abundance in stool (N) *CAG-448* abundance in stool (O) *CAG-488 sp000434055* abundance in stool (P) *CAG-632* abundance in stool (Q) *CAG-776 sp000438195* abundance in stool (R) *CAG-776* abundance in stool (S) *Comamonas B* abundance in stool (T) *Cyanobacteria* abundance in stool (U) *Ensifer* abundance in stool (V) *Escherichia flexneri* abundance in stool (W) *Escherichia* abundance in stool (X) *Faecalicoccus pleomorphus* abundance in stool (Y) *Gordonibacter* abundance in stool (Z) *Halarcobacter* abundance in stool (a) *Helicobacter* abundance in stool (b) *Hydrogenophaga* abundance in stool (c) *K10* abundance in stool (d) *KLE1615* abundance in stool (e) *Lachnospirales* abundance in stool (f) *Massiliomicrobiota* abundance in stool (g) *Olsenella C* abundance in stool (h) *Pandoraea* abundance in stool (i) *Photobacterium* abundance in stool (j) *Pseudomonadales* abundance in stool (k) *Raoultella* abundance in stool (l) *RUG420 sp900317985* abundance in stool (m) *Tepidanaerobacteraceae* abundance in stool (n) *UBA6960* abundance in stool (o) *UBA737* abundance in stool.


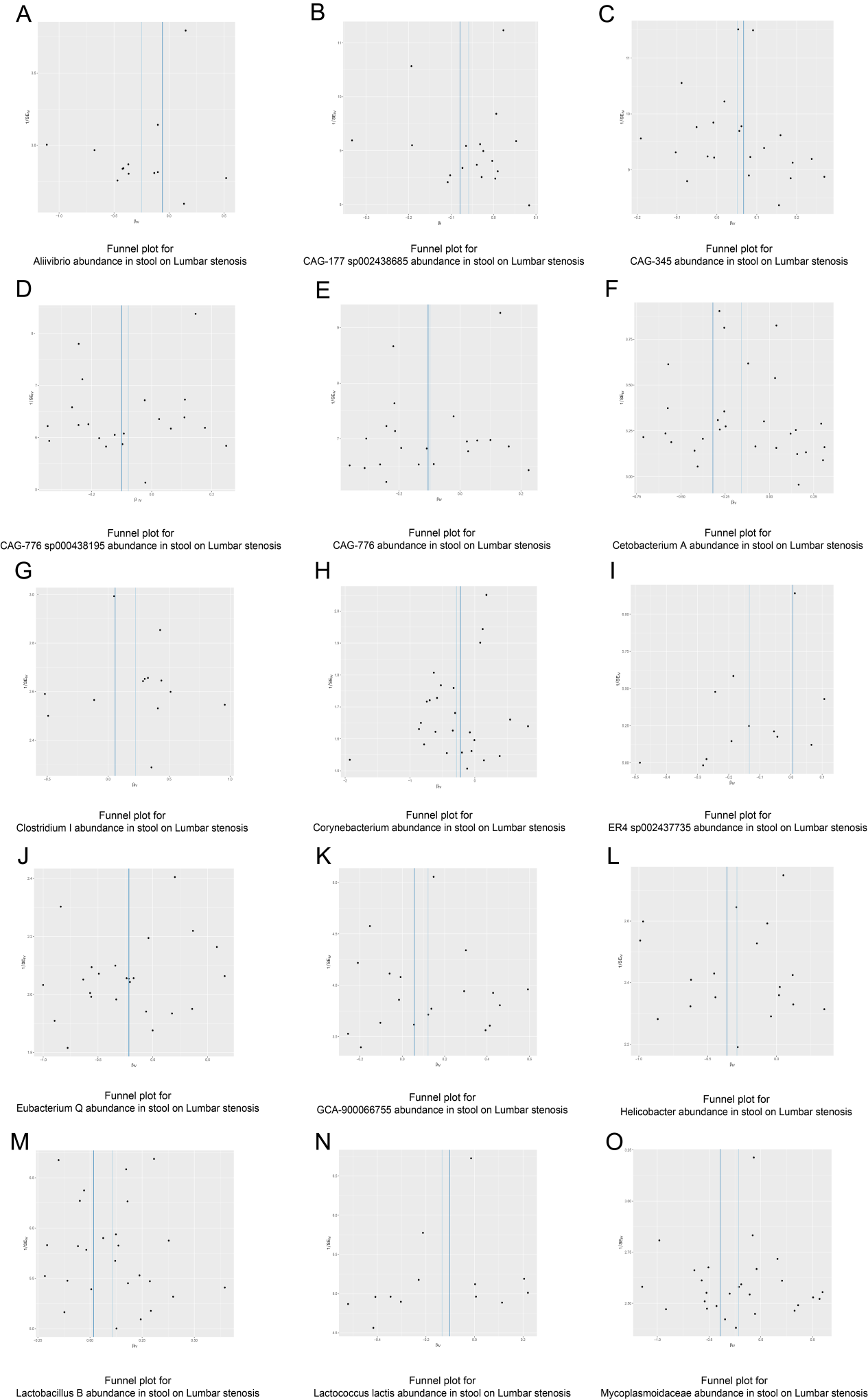


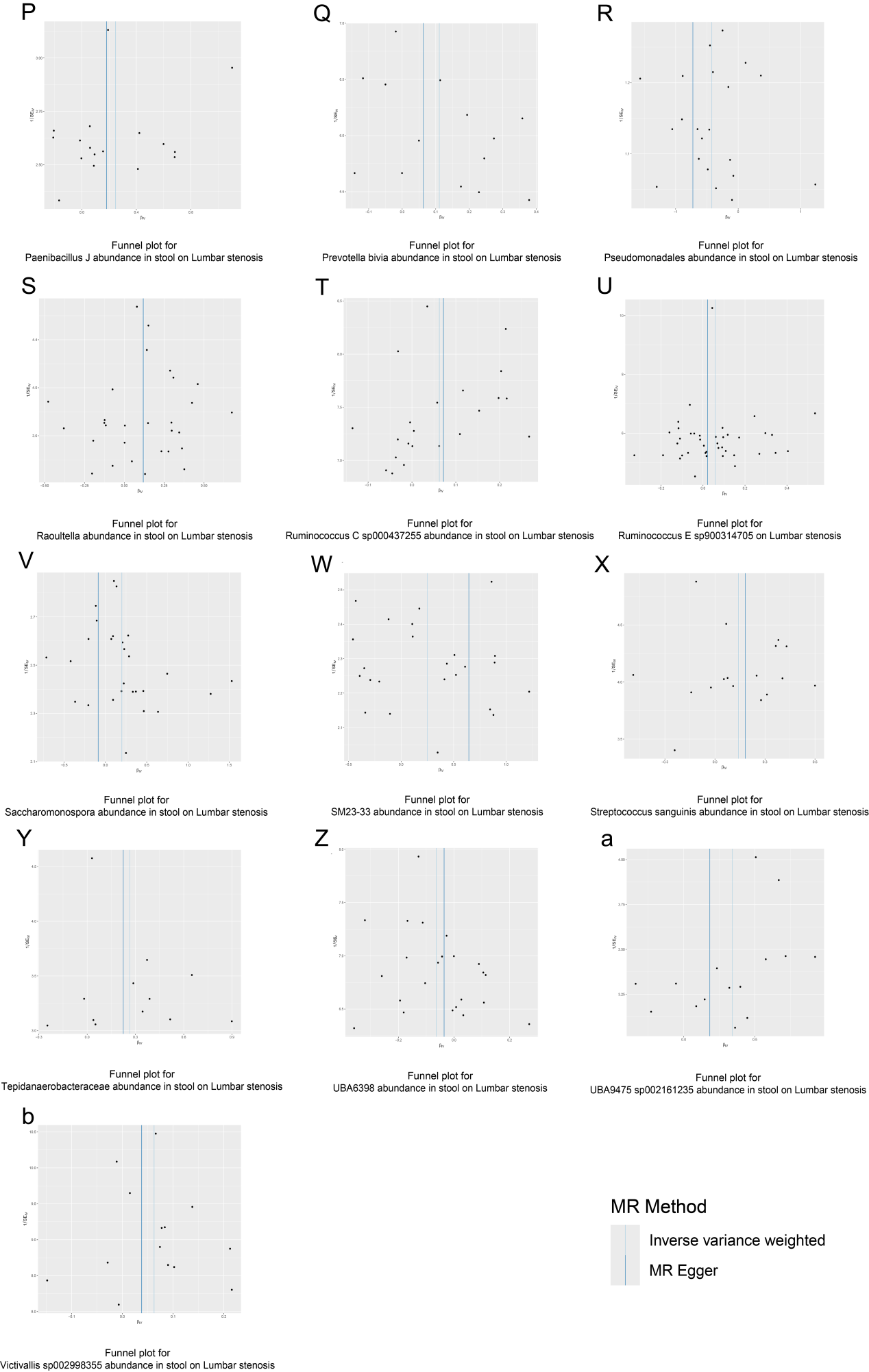


**Figure S4.** Funnel plot for gut microbiotas on lumbar stenosis. The analyses of (A) *Aliivibrio* abundance in stool (B) *CAG-177 sp002438685* abundance in stool (C) *CAG-345* abundance in stool (D) *CAG-776 sp000438195* abundance in stool (E) *CAG-776* abundance in stool (F) *Cetobacterium A* abundance in stool (G) *Clostridium I* abundance in stool (H) *Corynebacterium* abundance in stool (I) *ER4 sp002437735* abundance in stool (J) *Eubacterium Q* abundance in stool (K) *GCA-900066755* abundance in stool (L) *Helicobacter* abundance in stool (M) *Lactobacillus B* abundance in stool (N) *Lactococcus lactis* abundance in stool (O) *Mycoplasmoidaceae* abundance in stool (P) *Paenibacillus J* abundance in stool (Q) *Prevotella bivia* abundance in stool (R) *Pseudomonadales* abundance in stool (S) *Raoultella* abundance in stool (T) *Ruminococcus C sp000437255* abundance in stool (U) *Ruminococcus E sp900314705* abundance in stool (V) *Saccharomonospora* abundance in stool (W) *SM23-33* abundance in stool (X) *Streptococcus sanguinis* abundance in stool (Y) *Tepidanaerobacteraceae* abundance in stool (Z) *UBA6398* abundance in stool (a) *UBA9475 sp002161235* abundance in stool (a) *Victivallis sp002998355* abundance in stool.


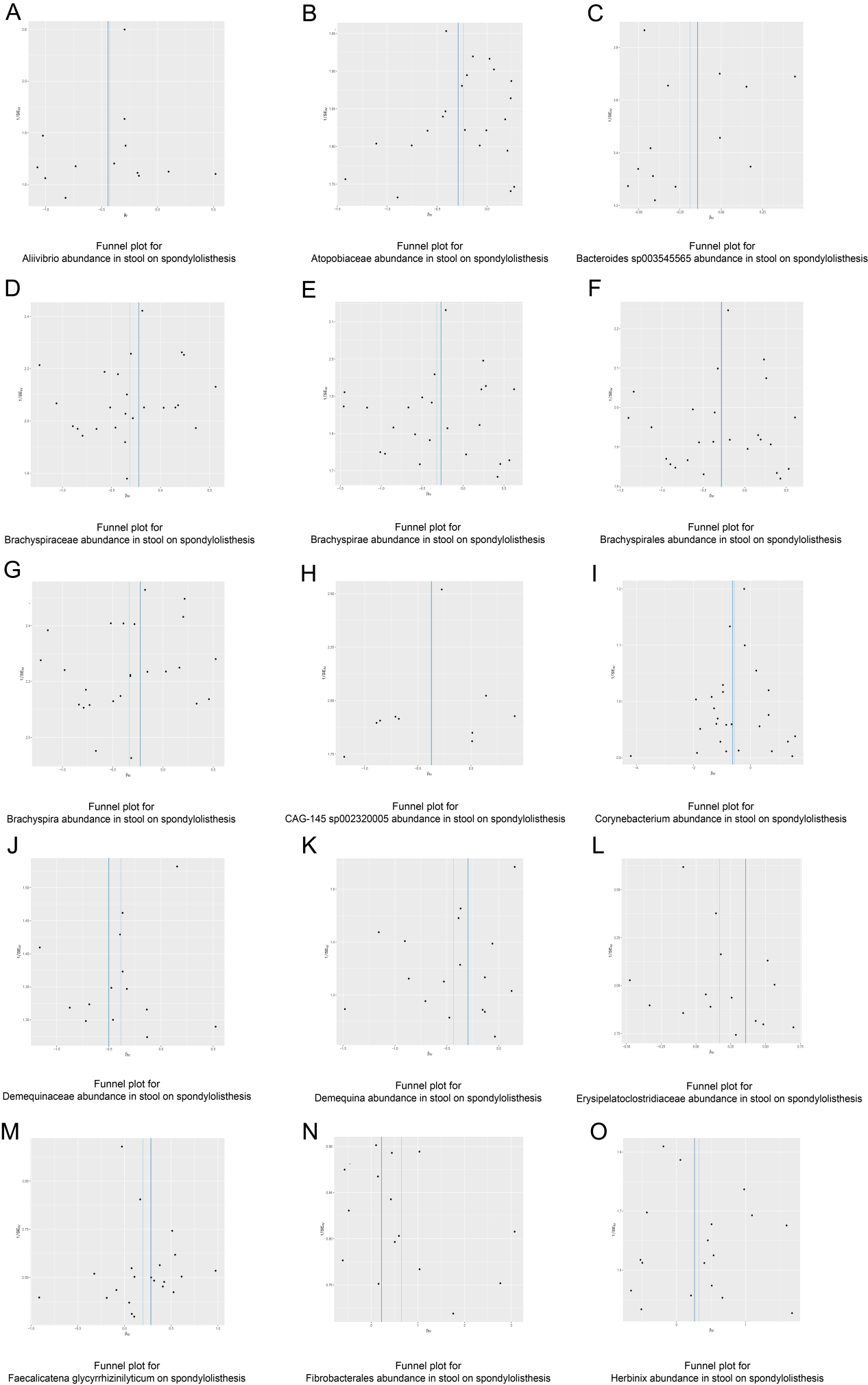


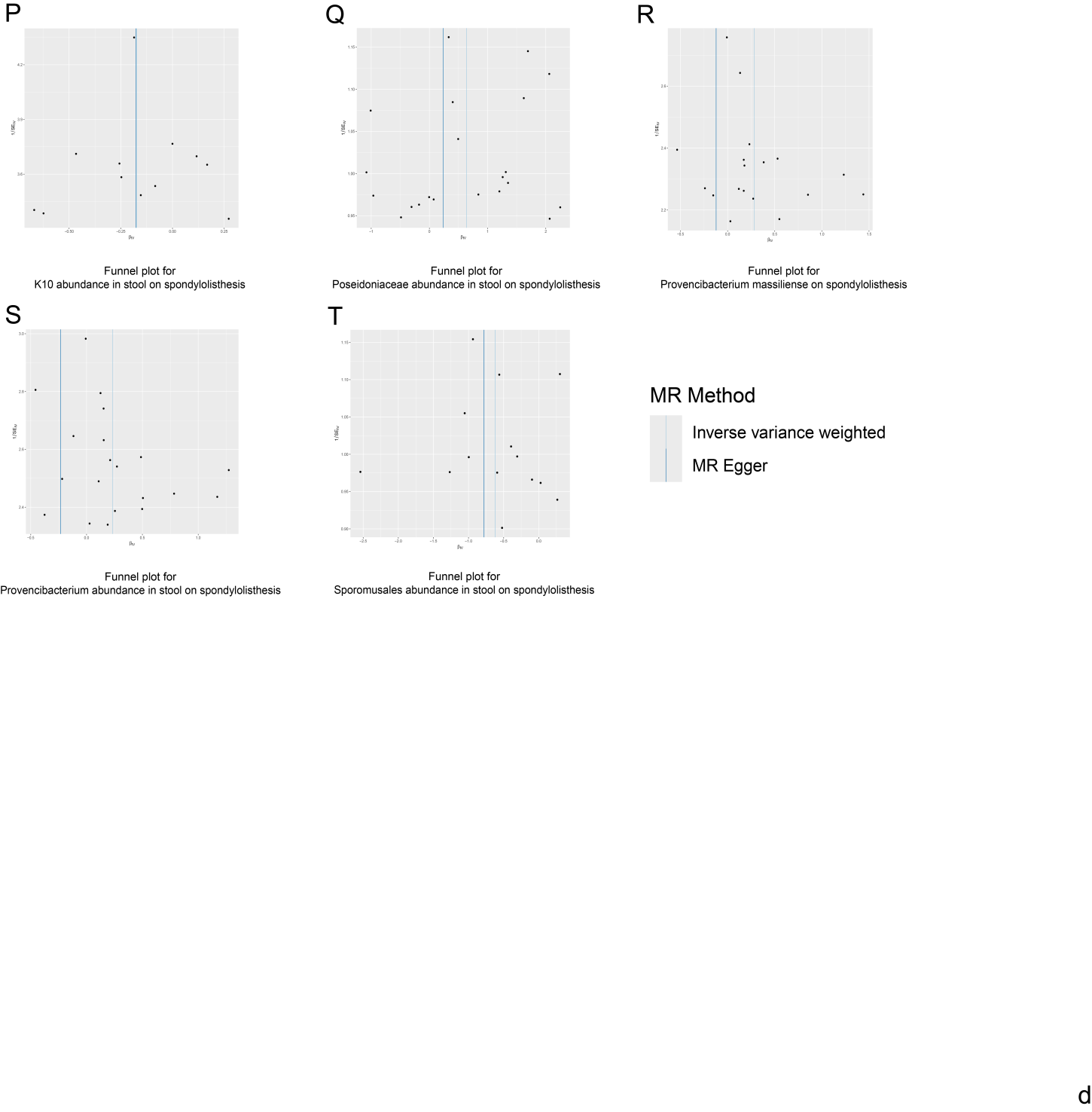


**Figure S5**: Funnel plot for gut microbiotas on spondylolisthesis. The analyses of (A) *Aliivibrio* abundance in stool (B) *Atopobiaceae* abundance in stool (C) *Bacteroides sp003545565* abundance in stool (D) *Brachyspiraceae* abundance in stool (E) *Brachyspirae* abundance in stool (F) *Brachyspirales* abundance in stool (G) *Brachyspira* abundance in stool (H) *CAG-145 sp002320005* abundance in stool (I) *Corynebacterium* abundance in stool (J) *Demequinaceae* abundance in stool (K) *Demequina* abundance in stool (L) *Erysipelatoclostridiaceae* abundance in stool (M) *Faecalicatena glycyrrhizinilyticum* abundance in stool (N) *Fibrobacterales* abundance in stool (O) *Herbinix* abundance in stool (P) *K10* abundance in stool (Q) *Poseidoniaceae* abundance in stool (R) *Provencibacterium massiliense* abundance in stool (S) *Provencibacterium* abundance in stool (T) *Sporomusales* abundance in stool.


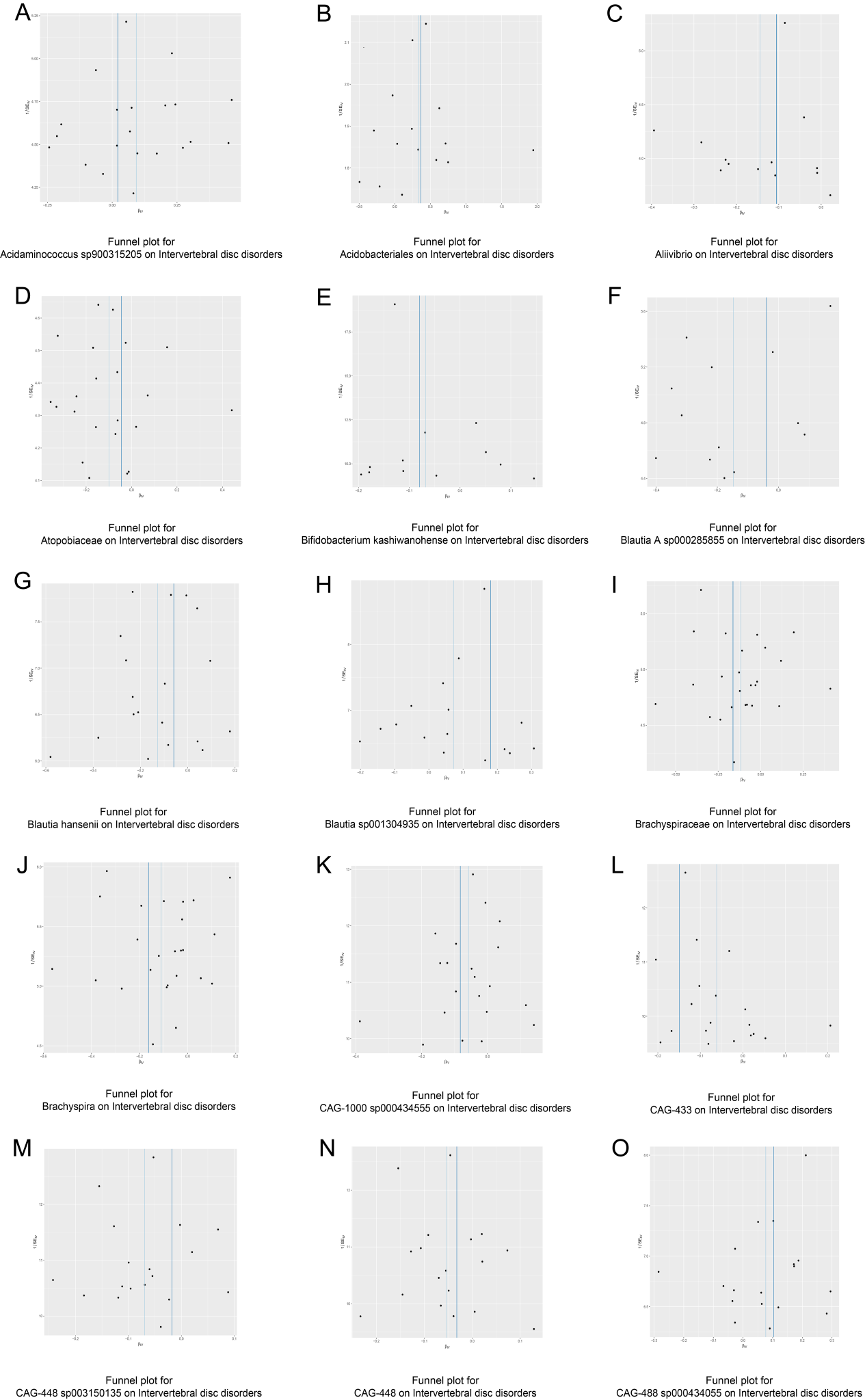


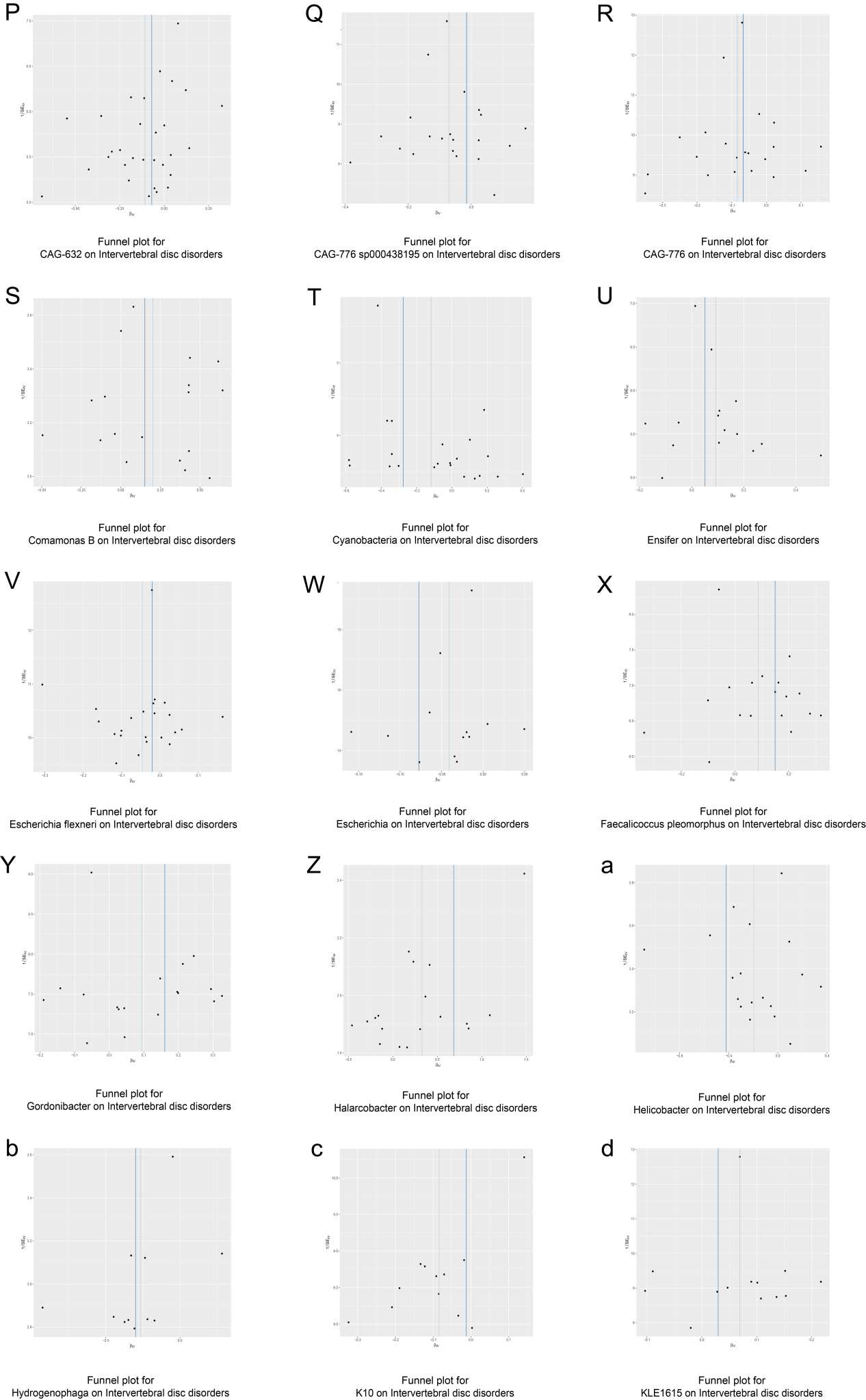


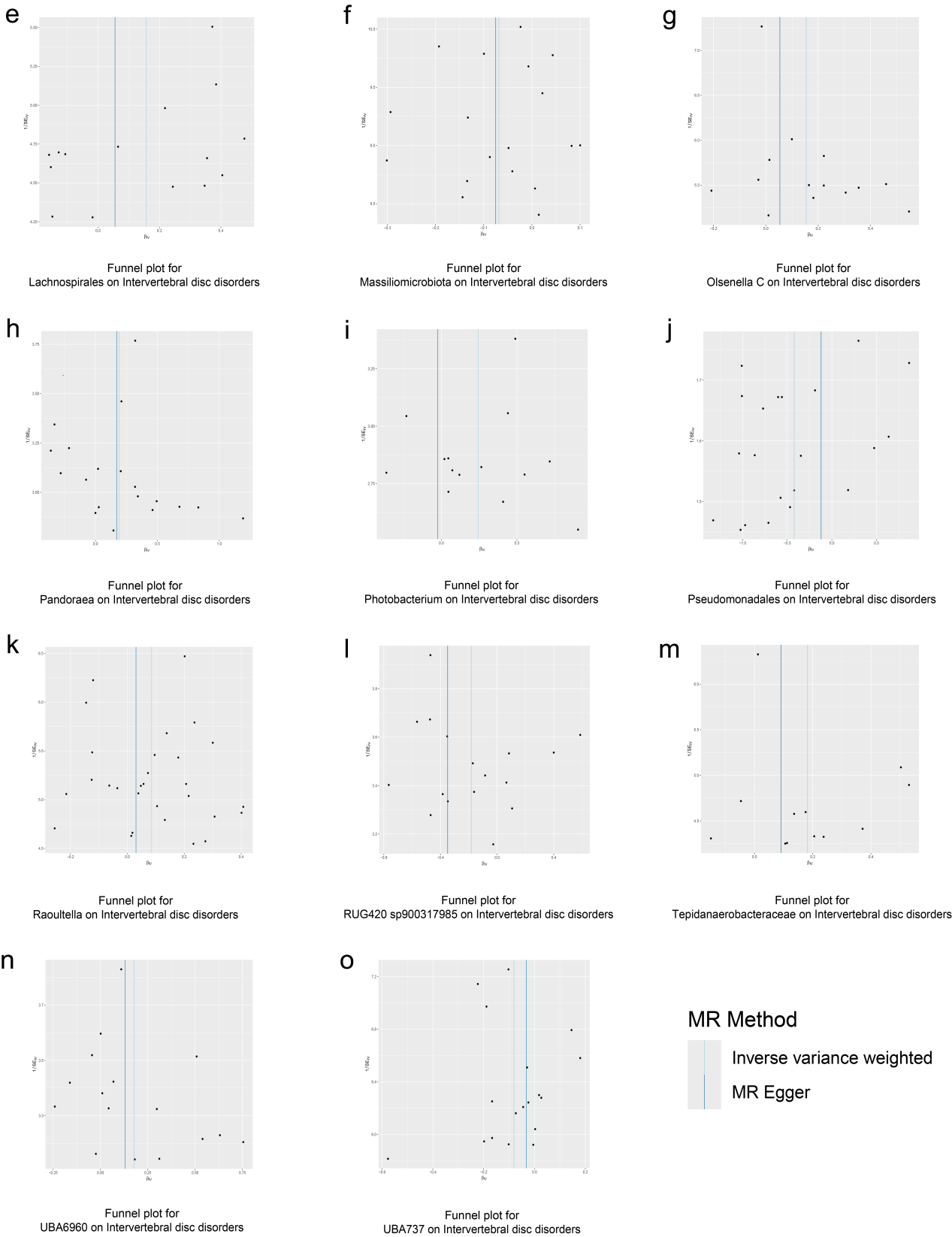


**Figure S6**: Funnel plot for gut microbiotas on intervertebral disc disorders. The analyses of (A) *Acidaminococcus sp900315205* abundance in stool (B) *Acidobacteriales* abundance in stool (C) *Aliivibrio* abundance in stool (D) *Atopobiaceae* abundance in stool (E) *Bifidobacterium kashiwanohense* abundance in stool (F) *Blautia A sp000285855* abundance in stool (G) *Blautia hansenii* abundance in stool (H) *Blautia sp001304935* abundance in stool (I) *Brachyspiraceae* abundance in stool (J) *Brachyspira* abundance in stool (K) *CAG-1000 sp000434555* abundance in stool (L) *CAG-433* abundance in stool (M) *CAG-448 sp003150135* abundance in stool (N) *CAG-448* abundance in stool (O) *CAG-488 sp000434055* abundance in stool (P) *CAG-632* abundance in stool (Q) *CAG-776 sp000438195* abundance in stool (R) *CAG-776* abundance in stool (S) *Comamonas B* abundance in stool (T) *Cyanobacteria* abundance in stool (U) *Ensifer* abundance in stool (V) *Escherichia flexneri* abundance in stool (W) *Escherichia* abundance in stool (X) *Faecalicoccus pleomorphus* abundance in stool (Y) *Gordonibacter* abundance in stool (Z) *Halarcobacter* abundance in stool (a) *Helicobacter* abundance in stool (b) *Hydrogenophaga* abundance in stool (c) *K10* abundance in stool (d) *KLE1615* abundance in stool (e) *Lachnospirales* abundance in stool (f) *Massiliomicrobiota* abundance in stool (g) *Olsenella C* abundance in stool (h) *Pandoraea* abundance in stool (i) *Photobacterium* abundance in stool (j) *Pseudomonadales* abundance in stool (k) *Raoultella* abundance in stool (l) *RUG420 sp900317985* abundance in stool (m) *Tepidanaerobacteraceae* abundance in stool (n) *UBA6960* abundance in stool (o) *UBA737* abundance in stool.


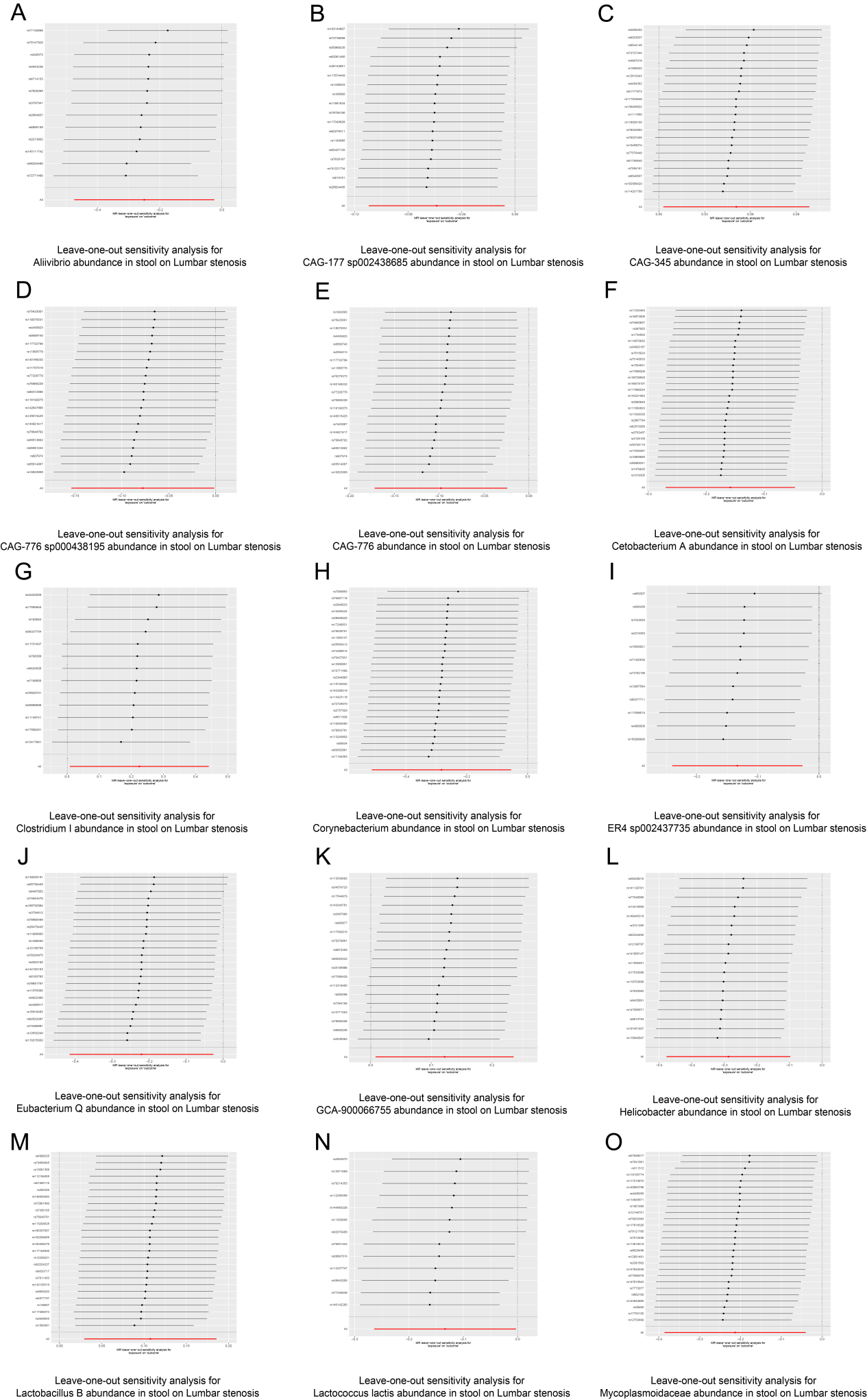


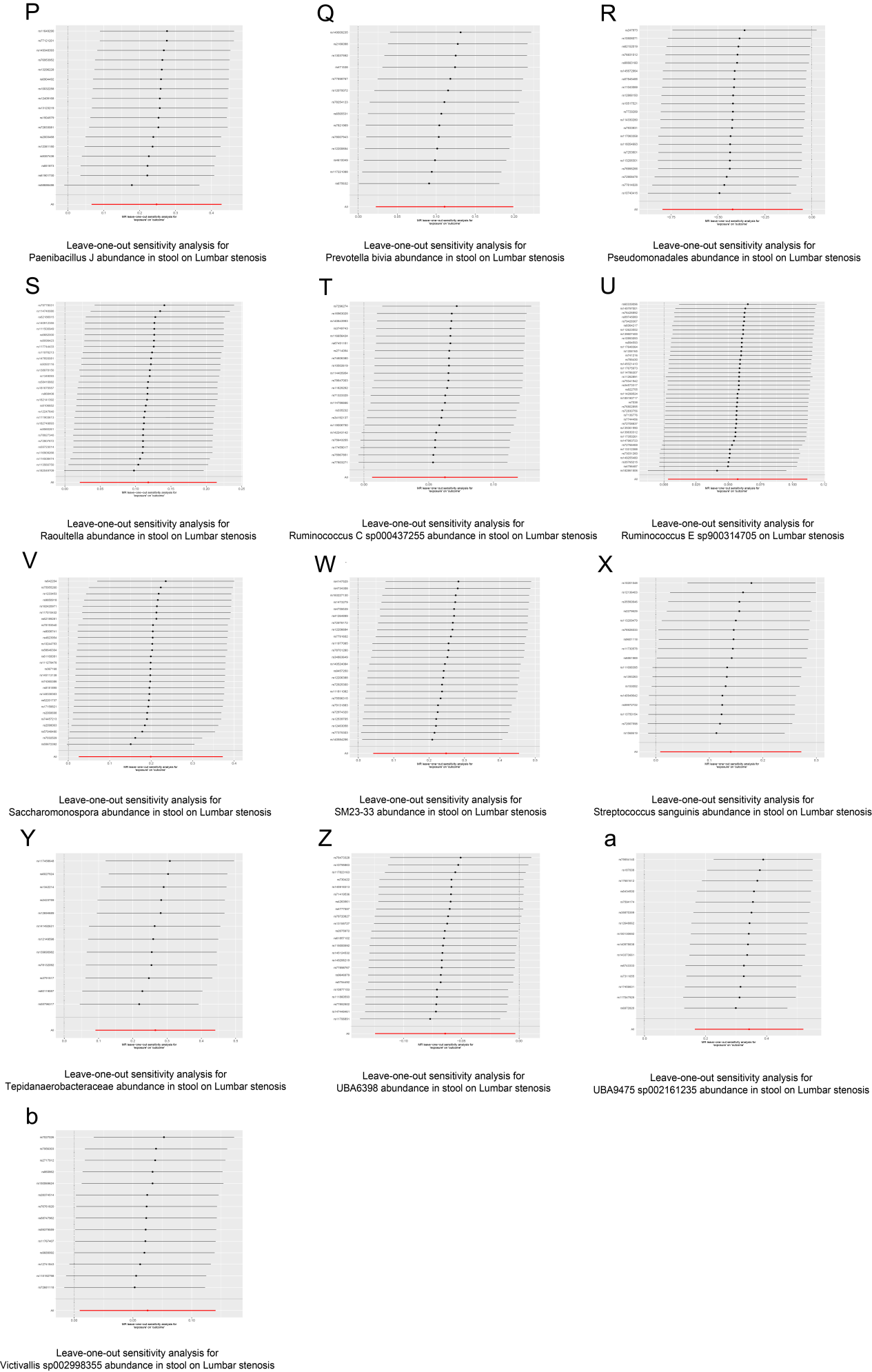


**Figure S7**: Leave-one-out analysis for gut microbiotas on lumbar stenosis. The analyses of (A) *Aliivibrio* abundance in stool (B) *CAG-177 sp002438685* abundance in stool (C) *CAG-345* abundance in stool (D) *CAG-776 sp000438195* abundance in stool (E) *CAG-776* abundance in stool (F) *Cetobacterium A* abundance in stool (G) *Clostridium I* abundance in stool (H) *Corynebacterium* abundance in stool (I) *ER4 sp002437735* abundance in stool (J) *Eubacterium Q* abundance in stool (K) *GCA-900066755* abundance in stool (L) *Helicobacter* abundance in stool (M) *Lactobacillus B* abundance in stool (N) *Lactococcus lactis* abundance in stool (O) *Mycoplasmoidaceae* abundance in stool (P) *Paenibacillus J* abundance in stool (Q) *Prevotella bivia* abundance in stool (R) *Pseudomonadales* abundance in stool (S) *Raoultella* abundance in stool (T) *Ruminococcus C sp000437255* abundance in stool (U) *Ruminococcus E sp900314705* abundance in stool (V) *Saccharomonospora* abundance in stool (W) *SM23-33* abundance in stool (X) *Streptococcus sanguinis* abundance in stool (Y) *Tepidanaerobacteraceae* abundance in stool (Z) *UBA6398* abundance in stool (a) *UBA9475 sp002161235* abundance in stool (a) *Victivallis sp002998355* abundance in stool.


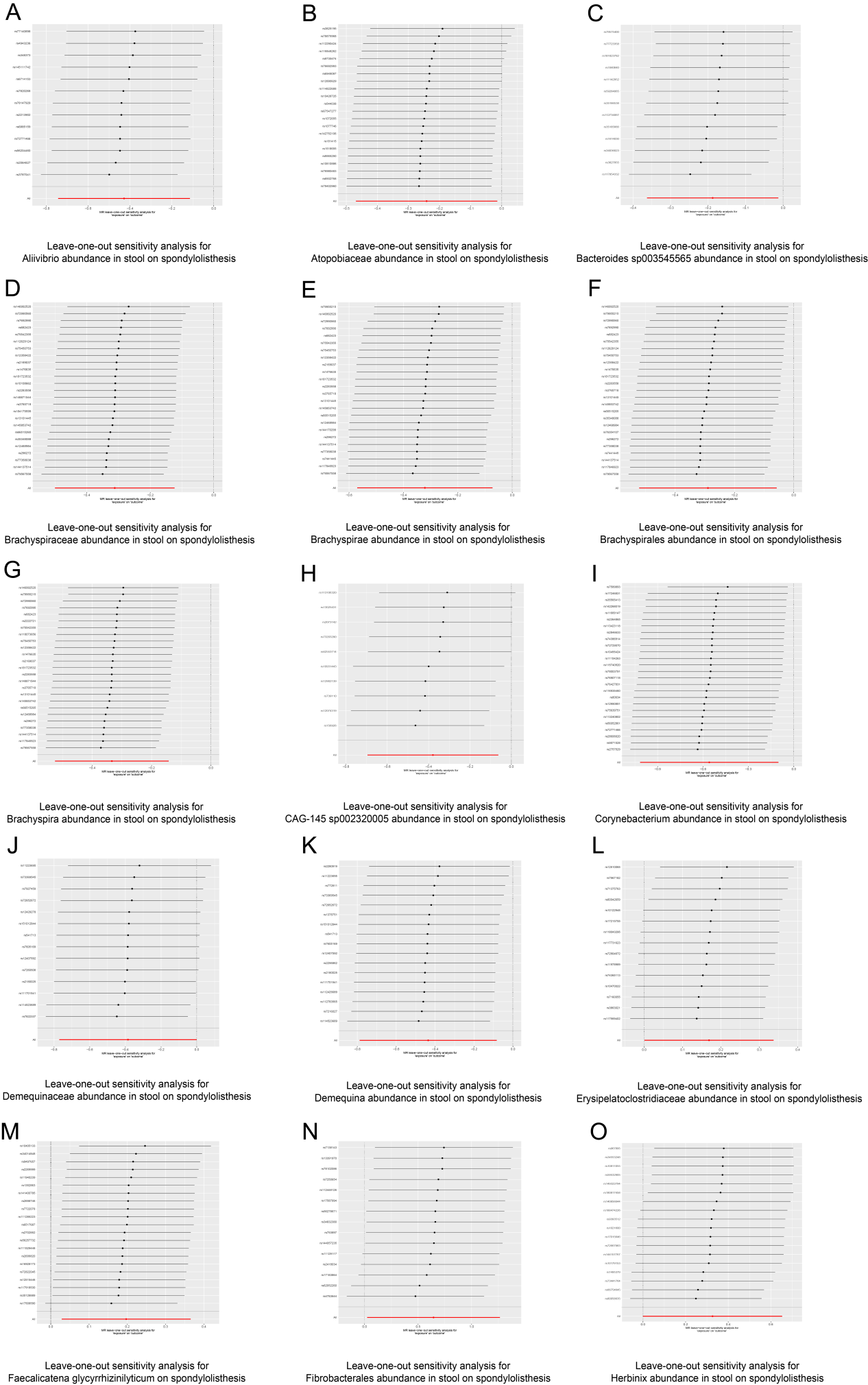


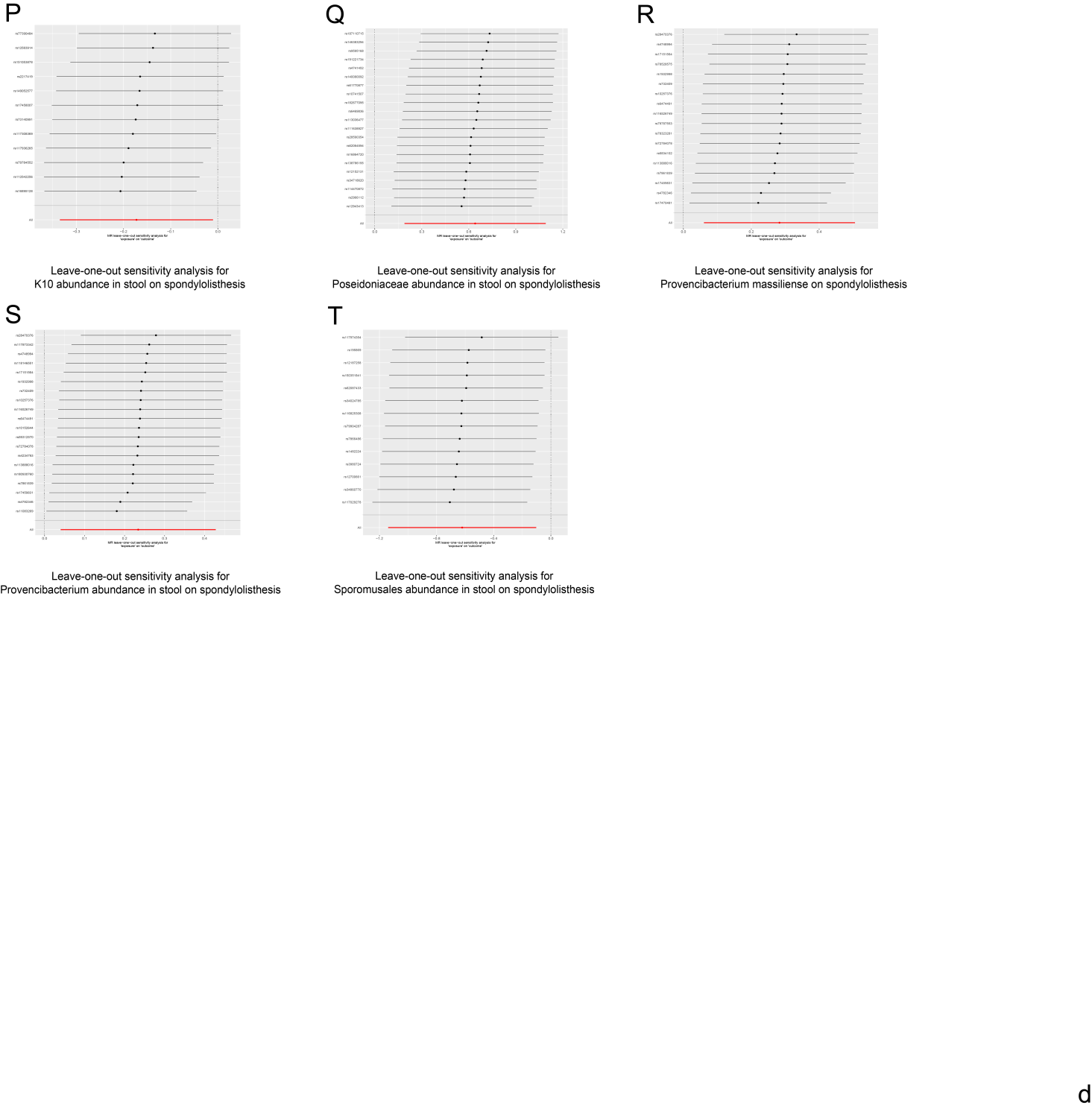


**Figure S8**: Leave-one-out analysis for gut microbiotas on spondylolisthesis. The analyses of (A) *Aliivibrio* abundance in stool (B) *Atopobiaceae* abundance in stool (C) *Bacteroides sp003545565* abundance in stool (D) *Brachyspiraceae* abundance in stool (E) *Brachyspirae* abundance in stool (F) *Brachyspirales* abundance in stool (G) *Brachyspira* abundance in stool (H) *CAG-145 sp002320005* abundance in stool (I) *Corynebacterium* abundance in stool (J) *Demequinaceae* abundance in stool (K) *Demequina* abundance in stool (L) *Erysipelatoclostridiaceae* abundance in stool (M) *Faecalicatena glycyrrhizinilyticum* abundance in stool (N) *Fibrobacterales* abundance in stool (O) *Herbinix* abundance in stool (P) *K10* abundance in stool (Q) *Poseidoniaceae* abundance in stool (R) *Provencibacterium massiliense* abundance in stool (S) *Provencibacterium* abundance in stool (T) *Sporomusales* abundance in stool.


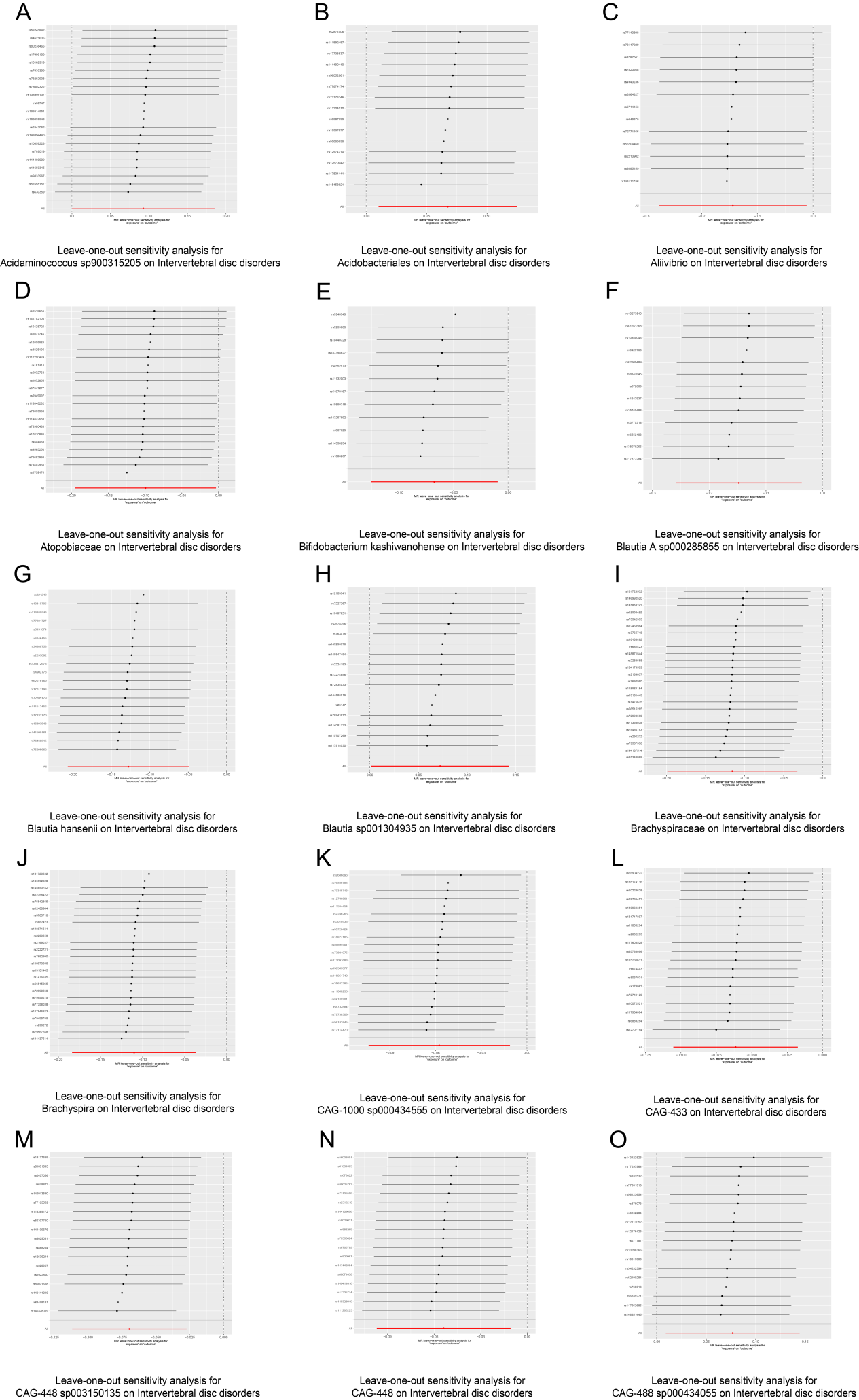


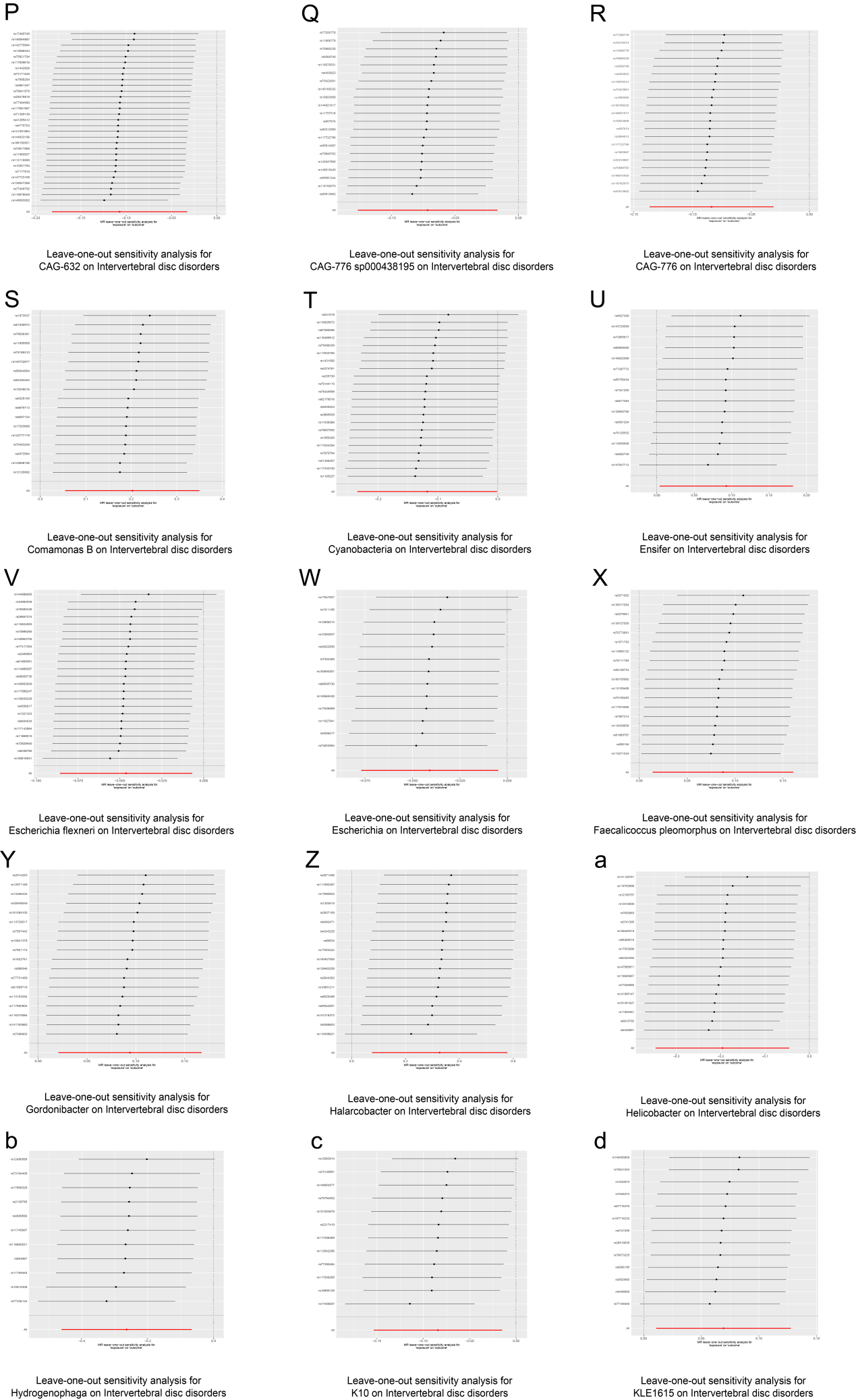


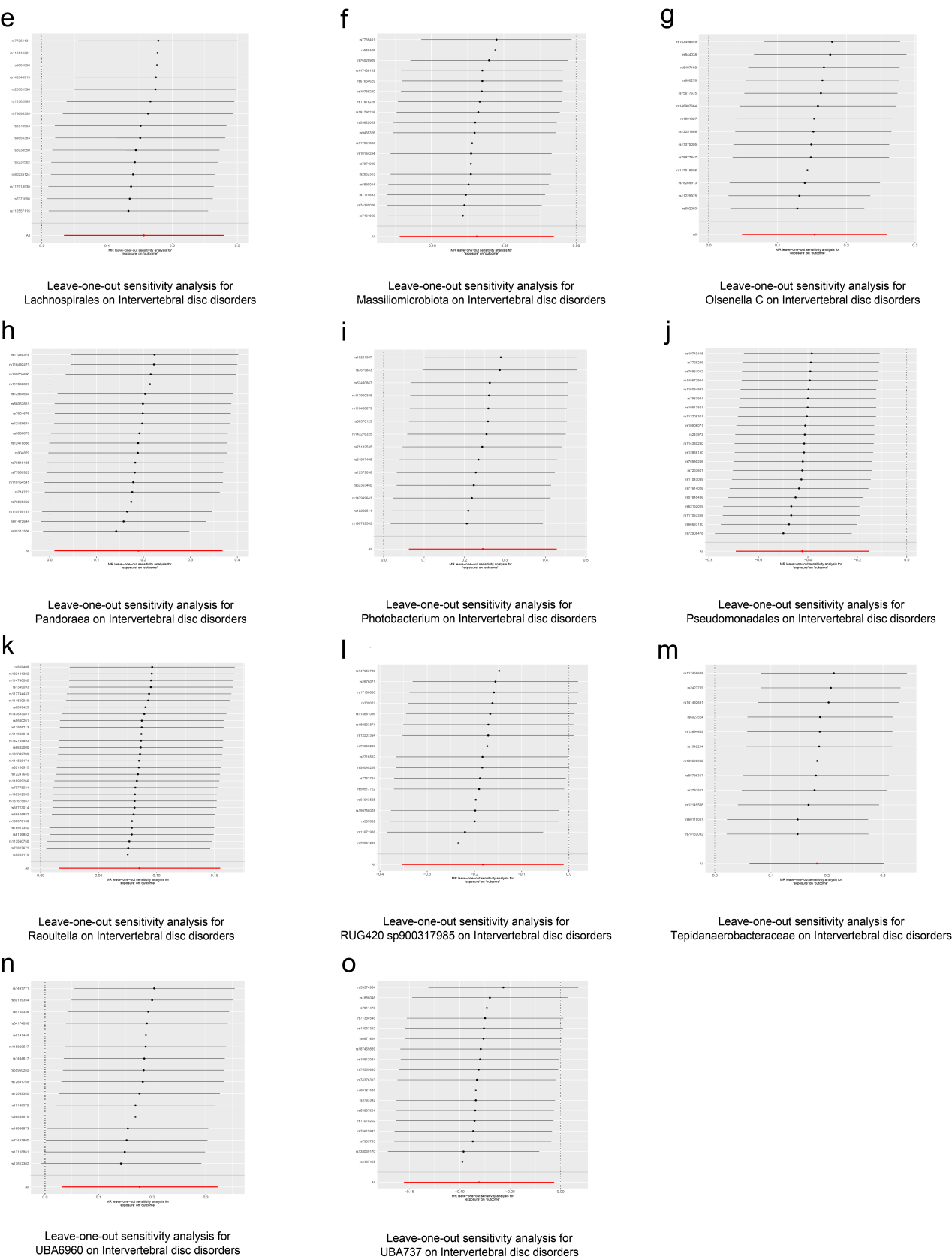


**Figure S9**: Leave-one-out analysis for gut microbiotas on intervertebral disc disorders. The analyses of (A) *Acidaminococcus sp900315205* abundance in stool (B) *Acidobacteriales* abundance in stool (C) *Aliivibrio* abundance in stool (D) *Atopobiaceae* abundance in stool (E) *Bifidobacterium kashiwanohense* abundance in stool (F) *Blautia A sp000285855* abundance in stool (G) *Blautia hansenii* abundance in stool (H) *Blautia sp001304935* abundance in stool (I) *Brachyspiraceae* abundance in stool (J) *Brachyspira* abundance in stool (K) *CAG-1000 sp000434555* abundance in stool (L) *CAG-433* abundance in stool (M) *CAG-448 sp003150135* abundance in stool (N) *CAG-448* abundance in stool (O) *CAG-488 sp000434055* abundance in stool (P) *CAG-632* abundance in stool (Q) *CAG-776 sp000438195* abundance in stool (R) *CAG-776* abundance in stool (S) *Comamonas B* abundance in stool (T) *Cyanobacteria* abundance in stool (U) *Ensifer* abundance in stool (V) *Escherichia flexneri* abundance in stool (W) *Escherichia* abundance in stool (X) *Faecalicoccus pleomorphus* abundance in stool (Y) *Gordonibacter* abundance in stool (Z) *Halarcobacter* abundance in stool (a) *Helicobacter* abundance in stool (b) *Hydrogenophaga* abundance in stool (c) *K10* abundance in stool (d) *KLE1615* abundance in stool (e) *Lachnospirales* abundance in stool (f) *Massiliomicrobiota* abundance in stool (g) *Olsenella C* abundance in stool (h) *Pandoraea* abundance in stool (i) *Photobacterium* abundance in stool (j) *Pseudomonadales* abundance in stool (k) *Raoultella* abundance in stool (l) *RUG420 sp900317985* abundance in stool (m) *Tepidanaerobacteraceae* abundance in stool (n) *UBA6960* abundance in stool (o) *UBA737* abundance in stool.


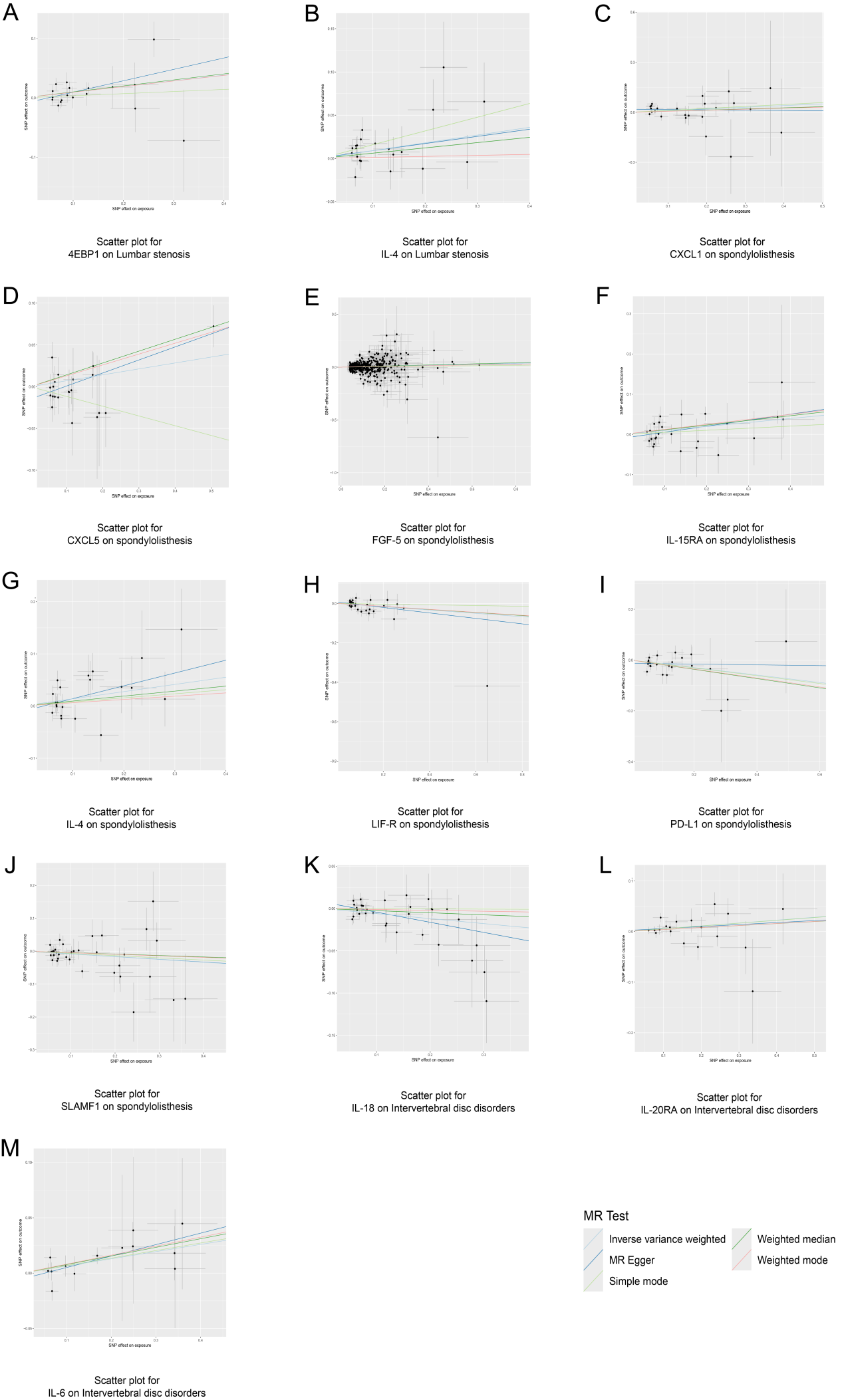


**Figure S10**: Scatter plot for inflammatory protein on lumbar diseases. The analyses of (A) 4EBP1 on lumbar stenosis (B) IL-4 on lumbar stenosis (C) CXCL1 on spondylolisthesis (D) CXCL5 on spondylolisthesis (E) FGF-5 on spondylolisthesis (F) IL-15RA on spondylolisthesis(G) IL-4 on spondylolisthesis (H) LIF-R on spondylolisthesis (I) PD-L1 on spondylolisthesis (J) SLAMF1 on spondylolisthesis (K) IL-18 on intervertebral disc disorders (L) IL-20RA on intervertebral disc disorders (M) IL-6 on intervertebral disc disorders.


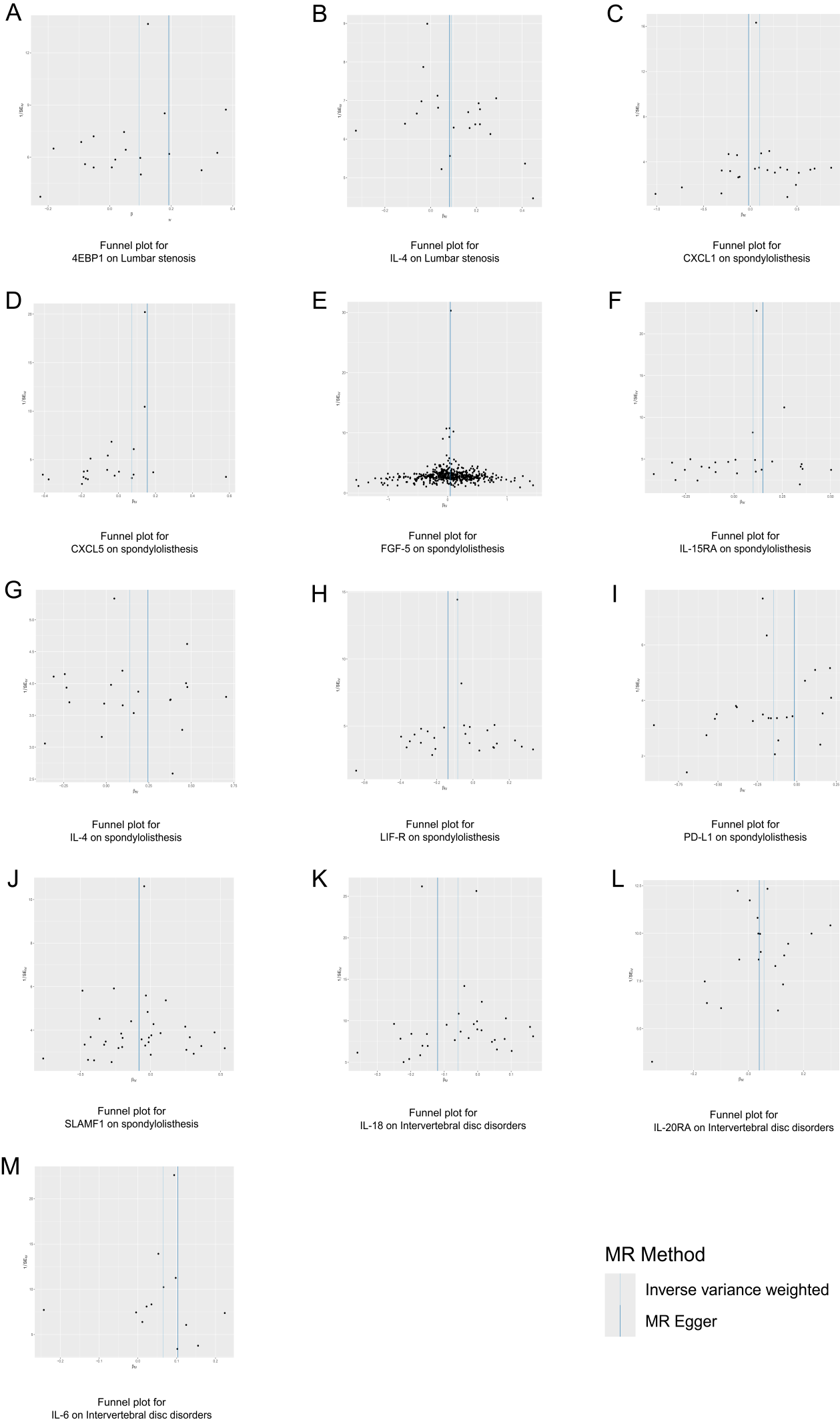


**Figure S11**: Funnel plot for inflammatory protein on lumbar diseases. The analyses of (A) 4EBP1 on lumbar stenosis (B) IL-4 on lumbar stenosis (C) CXCL1 on spondylolisthesis (D) CXCL5 on spondylolisthesis (E) FGF-5 on spondylolisthesis (F) IL-15RA on spondylolisthesis(G) IL-4 on spondylolisthesis (H) LIF-R on spondylolisthesis (I) PD-L1 on spondylolisthesis (J) SLAMF1 on spondylolisthesis (K) IL-18 on intervertebral disc disorders (L) IL-20RA on intervertebral disc disorders (M) IL-6 on intervertebral disc disorders.


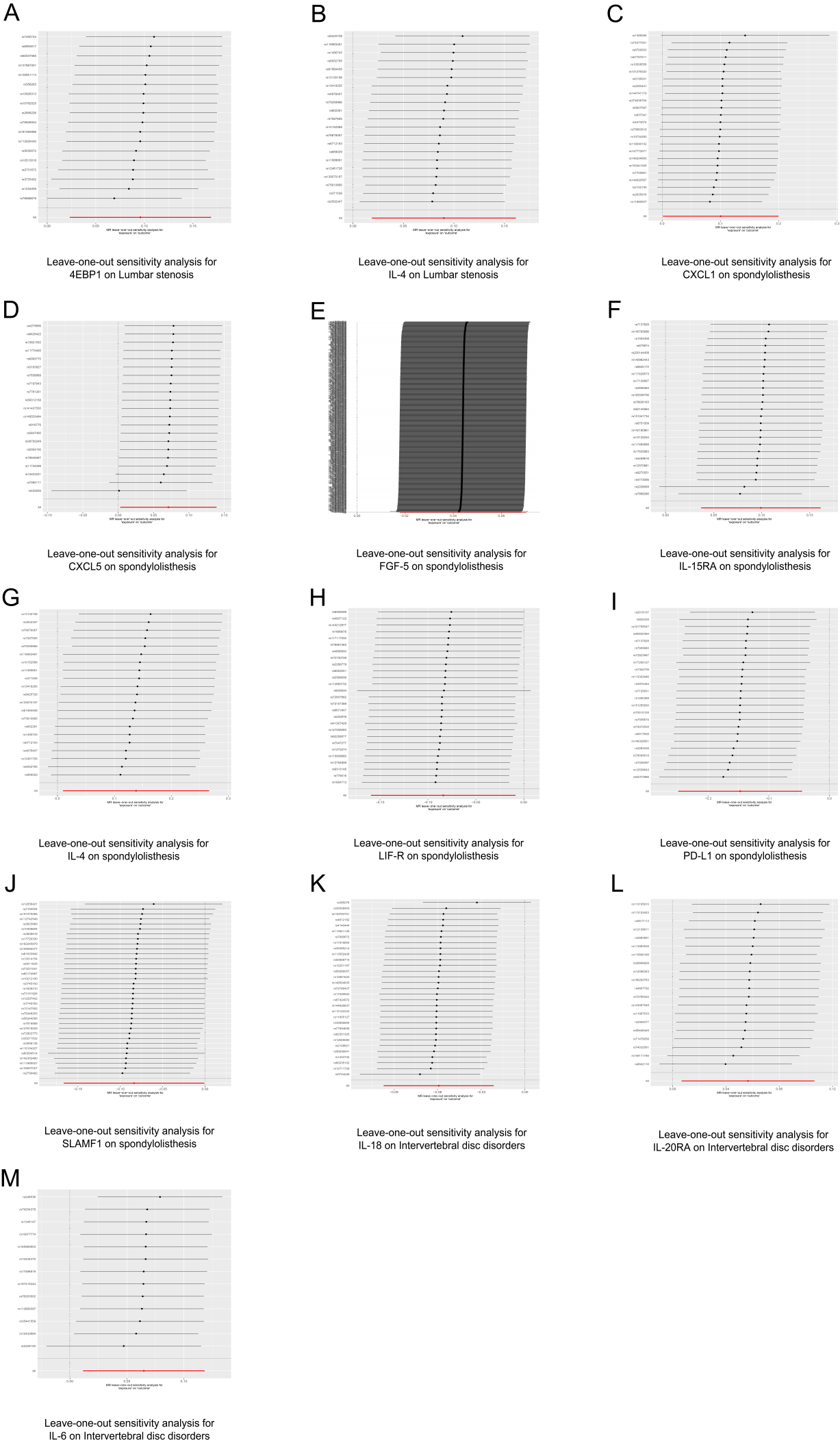


**Figure S12**: Leave-one-out analysis for inflammatory protein on lumbar diseases. The analyses of (A) 4EBP1 on lumbar stenosis (B) IL-4 on lumbar stenosis (C) CXCL1 on spondylolisthesis (D) CXCL5 on spondylolisthesis (E) FGF-5 on spondylolisthesis (F) IL-15RA on spondylolisthesis(G) IL-4 on spondylolisthesis (H) LIF-R on spondylolisthesis (I) PD-L1 on spondylolisthesis (J) SLAMF1 on spondylolisthesis (K) IL-18 on intervertebral disc disorders (L) IL-20RA on intervertebral disc disorders (M) IL-6 on intervertebral disc disorders.


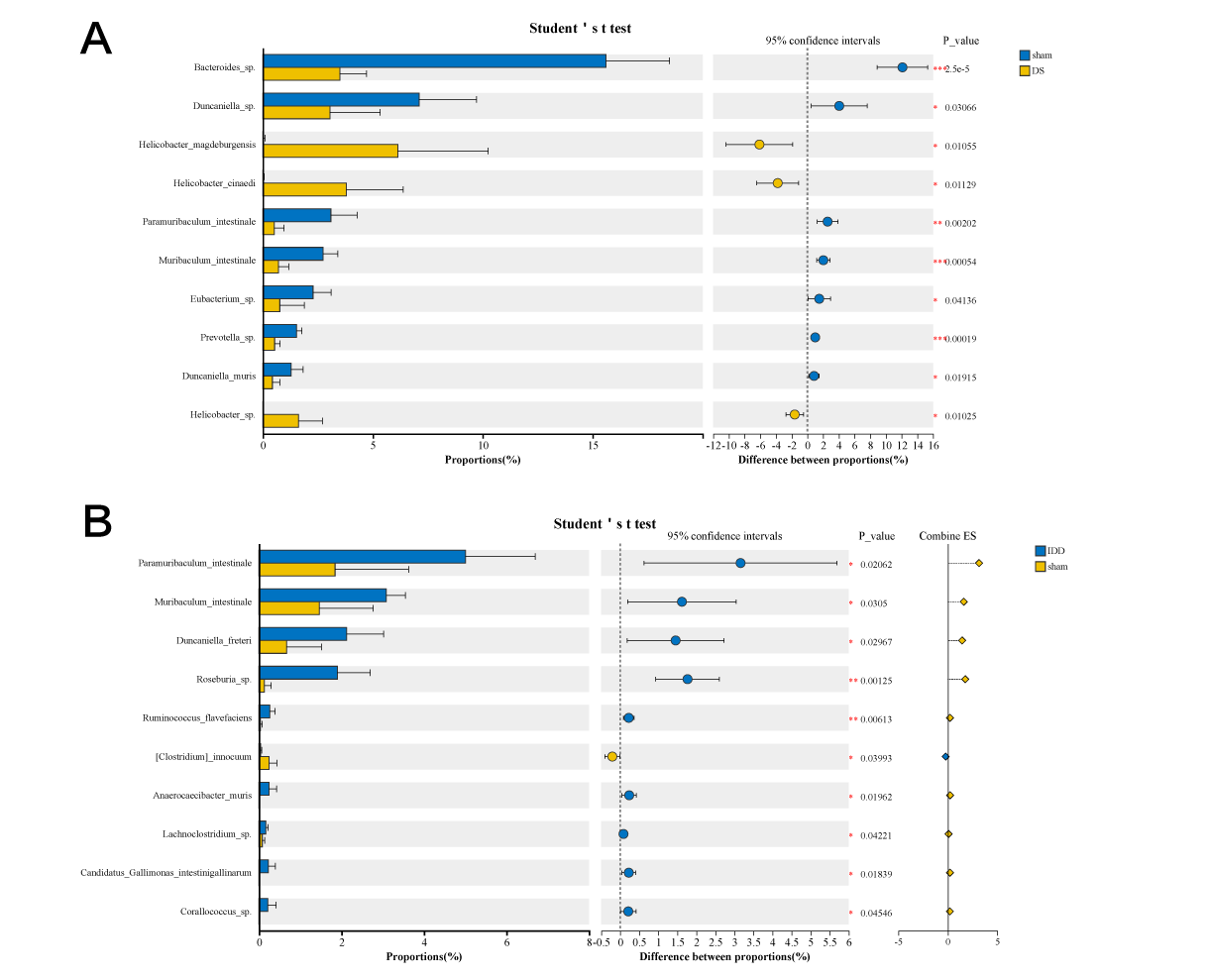


**Figure S13**: Gut microbiota sequencing analysis in rat models of lumbar spondylolisthesis and disc herniation. (A) lumbar spondylolisthesis (B) intervertebral disc disorders
